# A multimodal human-robot sign language interaction framework applied in social robots

**DOI:** 10.3389/fnins.2023.1168888

**Published:** 2023-04-11

**Authors:** Jie Li, Junpei Zhong, Ning Wang

**Affiliations:** ^1^School of Artificial Intelligence, Chongqing Technology and Business University, Chongqing, China; ^2^Department of Rehabilitation Sciences, The Hong Kong Polytechnic University, Kowloon, Hong Kong SAR, China; ^3^Bristol Robotics Laboratory, University of the West of England, Bristol, United Kingdom

**Keywords:** social robots, sign language, gesture recognition, multimodal sensors, human-robot interaction

## Abstract

Deaf-mutes face many difficulties in daily interactions with hearing people through spoken language. Sign language is an important way of expression and communication for deaf-mutes. Therefore, breaking the communication barrier between the deaf-mute and hearing communities is significant for facilitating their integration into society. To help them integrate into social life better, we propose a multimodal Chinese sign language (CSL) gesture interaction framework based on social robots. The CSL gesture information including both static and dynamic gestures is captured from two different modal sensors. A wearable Myo armband and a Leap Motion sensor are used to collect human arm surface electromyography (sEMG) signals and hand 3D vectors, respectively. Two modalities of gesture datasets are preprocessed and fused to improve the recognition accuracy and to reduce the processing time cost of the network before sending it to the classifier. Since the input datasets of the proposed framework are temporal sequence gestures, the long-short term memory recurrent neural network is used to classify these input sequences. Comparative experiments are performed on an NAO robot to test our method. Moreover, our method can effectively improve CSL gesture recognition accuracy, which has potential applications in a variety of gesture interaction scenarios not only in social robots.

## 1. Introduction

According to statistics, there are over 70 million deaf people in the world.^1^ For these people, communication with others through verbal language is impossible. Therefore, there are a great many difficulties in their daily communications. For instance, deaf people could not hear a horn when crossing the street. How to help the deaf community and those who have language impairment enjoy accessible social lives is very important. A service robot is a kind of intelligent robot dedicated to providing service for improving human life. With the development of robotics, information science, and sensor technology, service robots have been applied widely in many fields, such as medical rehabilitation, education, transportation, and entertainment to domestic service (Siciliano and Khatib, 2016; Yang et al., 2018a; Gonzalez-Aguirre et al., 2021). As a kind of service robot, the social robot is aimed at interacting with people in a human-centric way, which can provide a friendly way for interaction and services to meet the diverse demands of human beings (Breazeal et al., 2016; Yang et al., 2018b). Thus, social robots are expected to help the above-mentioned people communicate with others in a nonverbal way. In this sense, how to develop and design an intuitive, natural, easily interactive, and friendly interaction mode that can help these people communicate conveniently is a challenging topic for social robots.

Among various approaches to human-robot interaction (HRI), the way of using hand gestures for interaction facilitates more efficient communication between humans and robots. Since gesture interaction is a kind of non-contact way, which is more secure, friendly, and easy to accept by humankind. The gesture is one of the most widely used communicative manners. In the long-term social practice process, the gesture is endowed with a variety of specific meanings. At present, gesture has become the most powerful tool for expressing sentiment, intention, or attitude for humans. Hence, more and more researchers focus on gesture recognition and its applications. Many approaches are studied to recognize hand gestures by different modality sensors with various features. These approaches can be mainly categorized into three types: the wearable sensor-based approaches (Si et al., 2022), the vision sensor-based approaches (Mitra and Acharya, 2007; Oudah et al., 2020; Rastgoo et al., 2020; AI Farid et al., 2022), and the combination of the above-mentioned gesture recognition approaches (Wu et al., 2016; Xue et al., 2018; Roda-Sanchez et al., 2023). However, most of these studies were based on the single static or dynamic gestures to classification or recognition. Seldom of them focused on both dynamic and static recognition by using different modal information. Dynamic and static gestures are both needed to recognize under some specific circumstances, such as sign language recognition (SLR) for deaf or speech-impaired people.

Sign language is highly structural hand gestures, including static gestures and dynamic gestures. It serves as a useful tool for the deaf and hearing-impaired individuals in daily communication. The structural features of sign language make it very suitable for computer vision algorithms (Wu and Huang, 1999). Therefore, many relevant studies (such as SLR) are based on vision-based approaches (Cheok et al., 2019). The input data of vision-based SLR algorithms are usually divided into static gesture and dynamic gesture. Correspondingly, there are static-based and dynamic-based SLR approaches. For static sign language gestures, the approaches, such as K-nearest neighbor (Tharwat et al., 2015), support vector machine (Kurdyumov et al., 2011), and multilayer perceptron (Karami et al., 2011) are used to obtain better results. The vision-based dynamic sign language approaches include hidden Markov model (HMM; Wang et al., 2003), dynamic time wrapping (Lichtenauer et al., 2008), relevance vector machine (Wong and Cipolla, 2005), and finite state machine (Hong et al., 2000), etc.

Recently, with the advent of deep neural networks (DNN, Cao et al., 2022a), various deep learning algorithms are applied to SLR (Camgoz et al., 2018; Cui et al., 2019; Qi et al., 2021). Pu et al. presented a dynamic convolutional neural network (CNN) SLR model based on RGB video input (Pu et al., 2018). Wei et al. combined the 3D convolutional residual network and bidirectional long short-term memory (LSTM) network to recognize dynamic sign language gestures (Wei et al., 2019). Similarly, Cui et al. developed a dynamic SLR framework by combining CNN and bidirectional LSTM networks (Cui et al., 2019). Ye et al. proposed a 3D Recurrent CNN to classify gestures and localize joints (Ye et al., 2018). With the development of sensor technology (Chen et al., 2020; Cao et al., 2022b), many high accuracy and low cost sensors appears, such as Kinect and Leap Motion Controller (LMC) sensors. These sensors can capture hand or arm information more conveniently. The combination of new emerging sensors and deep learning approaches brings more new possibilities for SLR. Chong and Lee used the features recorded from the LMC sensor to classify 26 letters in American Sign Language (ASL). The recognition accuracy reaches 93.81% with DNN algorithms (Chong and Lee, 2018). Naglot et al. used a deep learning method to achieve 96.15% based on LMC gesture samples (Naglot and Kulkarni, 2016). Kumar et al. (2017a) presented a multimodal framework combining the HMM and bidirectional LSTM networks. The framework can recognize isolated sign language gesture datasets from Kinect and LMC sensors. To improve the accuracy of SLR, researchers fused different features to achieve the expected results. Kumar et al. (2017a) classified 25 Indian sign language (ISL) gestures by employing the coupled HMM to fuse the Leap Motion and Kinect sign language information. Bird et al. (2020) presented a late fusion approach to multimodality in SLR by fusing RGB and 3D hand data with a deep convolutional network. In the above research works, the sign language gestures involve both isolated static and dynamic hand gestures, based on Chinese sign language (CSL), ISL, ASL, and other sign languages from different countries, etc. The SLR approaches include traditional machine learning, deep learning, and the combination of both algorithms. However, these studies seldom take into account both static and dynamic sign language gestures in a classifier at the same time. Moreover, most of the researchers focus on using depth or RGB information as the input data of the classifier. Generally, the fusion of different modal input data also often uses these two data. The SLR framework proposed by Bird et al. fused two modalities of gesture datasets both captured from one sensor (LMC; Bird et al., 2020). Hence, inspired by the previous work (Naglot and Kulkarni, 2016; Kumar et al., 2017a,b; Bird et al., 2020), we propose a multimodal SLR framework that combines CSL features from several sensors to recognize static and dynamic hand gestures. This framework uses the deep learning method to fuse two modalities features from two different sensors to improve the recognition accuracy. The proposed multimodal framework can not only recognize singular CSL gestures but also recognize gestures consisting of two singular gestures.

Sign language mainly use the human hands to convey information. In some cases, other body parts such as fingers, arms, and head also used to convey information (Wu and Huang, 1999). CSL gestures mainly use human hands and arms. Therefore, the focus of this paper is to use human hand and arm information to classify corresponding gestures. Different from most of the vision-based input data, this work fuses the information from the visual sensor and surface electromyography (sEMG) signals by using Leap Motion and a wearable Myo armband. Though some research works in the gesture recognition area use human arm sEMG captured by Myo armband or other similar devices. Sometimes, higher recognition accuracy is also achieved. But for SLR, seldom research applies sEMG signals to classify different sign language gestures. In this work, considering the characteristic of CSL, we apply the advantages of information fusion to fuse two modalities of data to improve recognition accuracy. It combines the advantages of arm sEMG information in gesture recognition and the complementary for different modal sensor information. Besides, SLR is mainly applied to the daily communication between deaf, speech-impaired, and autism spectrum disorders (ASD) communities, the proposed CSL recognition framework is applied to social robots. Thus, it can promote communication between these communities and entertainment with robots.

The main contribution of this work is that an HRI system by integrating two modalities of CSL data is developed for deaf and speech-impaired people, which enables the social robots to communicate with target people efficiently and friendly. Most importantly, the proposed system can be applied in other interaction scenarios between robots and autistic children. The remainder of the paper is organized as follows. The CSL gestures classification method is presented in section 2. Section 3 provides the simulations and case studies on the real-world robot. Section 4 concludes this work and discusses the further potential applications.

## 2. Methodology

**Figure 1:**
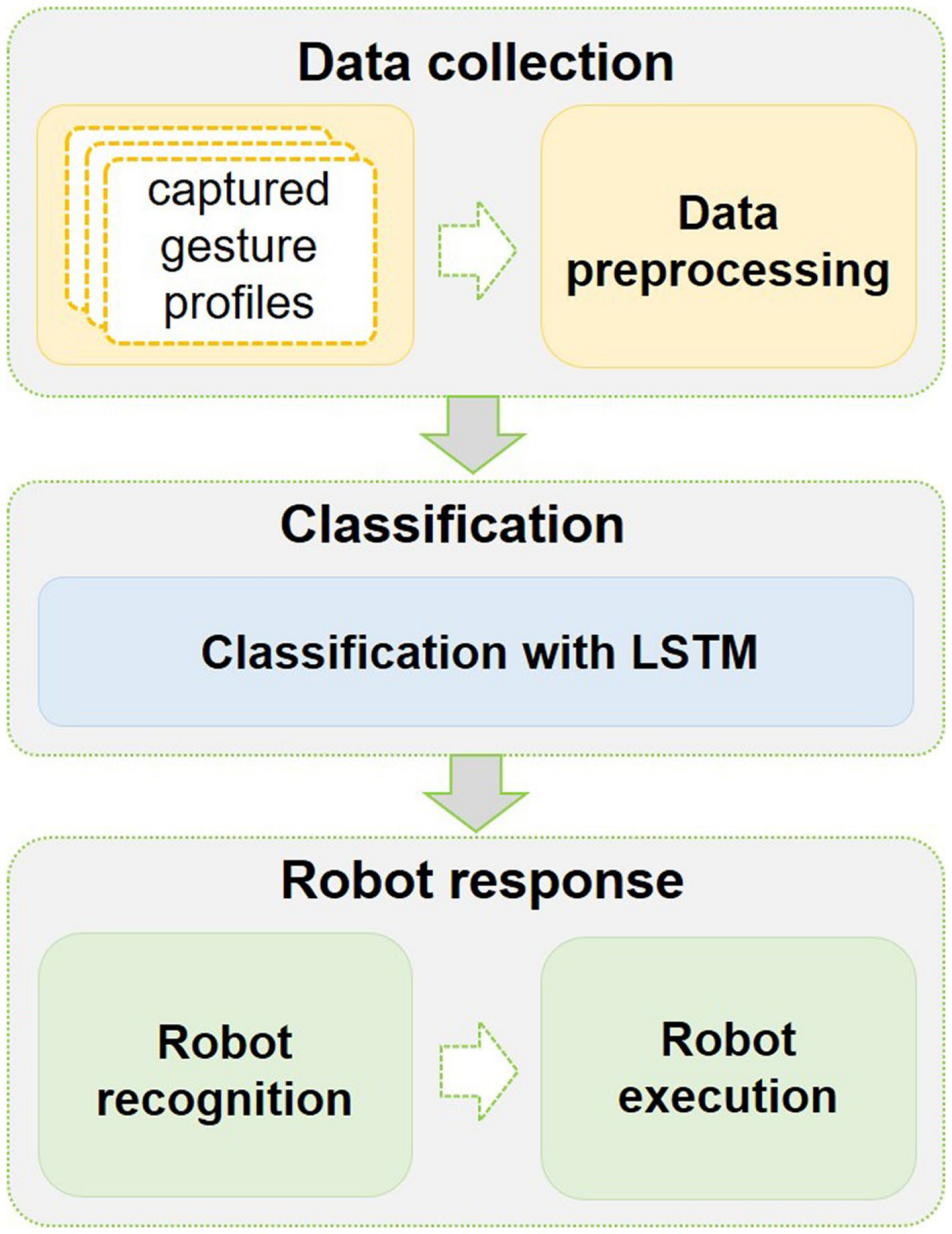
The framework of the proposed multimodal human-robot interaction (HRI) system.

The overview of the proposed HRI system is shown in Figure 1. It includes three phases: data collection, data classification, and robot response.

### 2.1. System overview

In this data collection phase, we mainly collect four different kinds of common CSL gestures. Here, two modalities of hand action data are collected from two different modal sensors. Leap Motion is applied to capture human hand 3D features. Meanwhile, the human arm sEMG signals are captured by the Myo armband.

In the data classification phase, the collected gesture data from two sensors are preprocessed, respectively. Then, the features of two modalities datasets are fused as one dataset, which serves as input to the LSTM classifiers.

In the robot response phase, after the gesture data is recognized by the LSTM classifier, the results are transformed into executable commands of the social robot. Later, the robot makes a response to the recognition results.

### 2.2. Data collection and preprocessing

**Figure 2:**
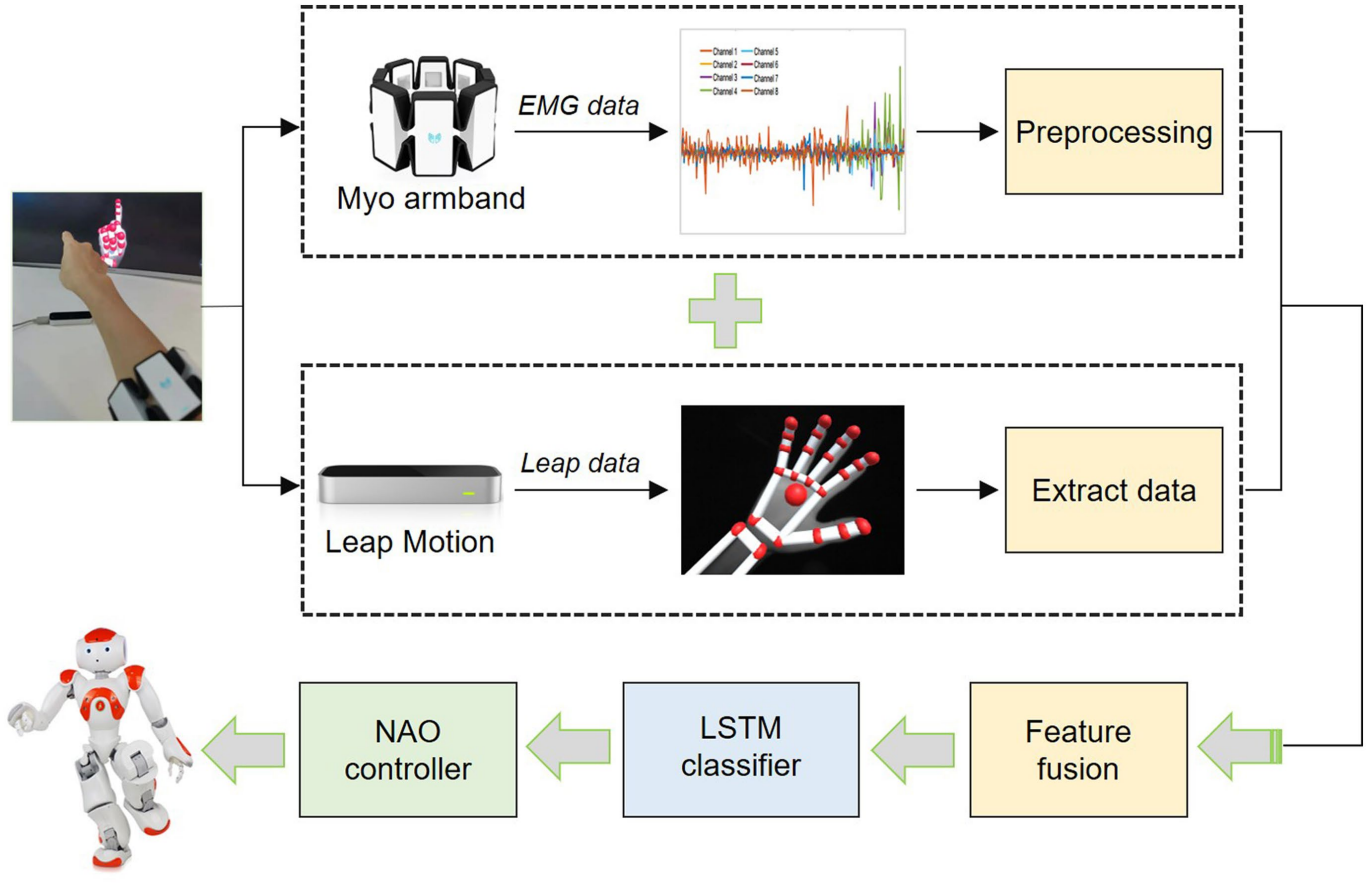
An overall diagram of the HRI system.

Figure 2 presents the overall steps of the data collection, preprocessing, and feature fusion. As aforementioned, the Myo armband and LMC are used to capture arm sEMG signals and human hand movements, respectively. As shown in Figure 2, a participant wears the Myo armband on the forearm and puts his/her hand onto the Leap Motion sensor within viewing range to capture sEMG signals and hand movement information synchronously. When a participant is performing a certain sign language, the data are recorded synchronously from the Myo armband and LMC. That is, the sEMG signals and human hand 3D vectors from both sensors are timely collected. In this paper, four daily CSL gestures are considered.

#### 2.2.1. Hand 3D information captured by leap motion sensor

**Figure 3:**
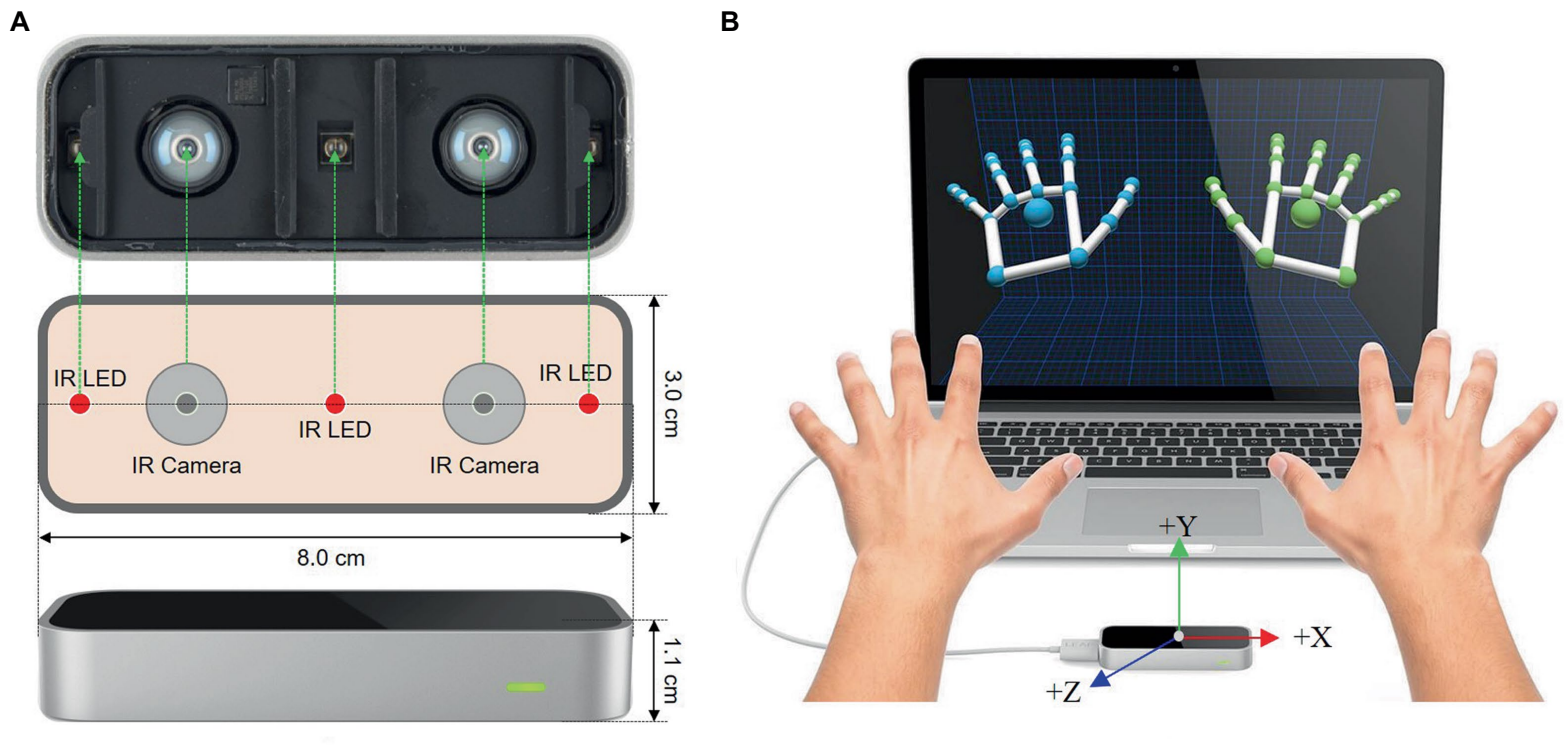
The view of Leap Motion Controller (LMC). **(A)** Schematic view of LMC. **(B)** 3D view of human hand from LMC (Weichert et al., 2013).

Leap motion is an optical hand tracking sensor that captures the movements of human hands with sub-millimeter accuracy. The sketch of LMC is shown in Figure 3A. The core of the device consists of three infrared LEDs placed at equal distances from each other, and two stereo cameras placed between each pair of IR sensors (Li et al., 2019). With these devices, LMC can detect the bones and joints of the human hand accurately by combining stereoscopy and depth-sensing. The view of a 3D representation of the hand translated by the two cameras is shown in Figure 3B. Compared with the Microsoft Kinect sensor, LMC is more portable, smaller ($L\times W\times H=8\times 3\times 1.1\;{\mathrm{cm}}^{3}$), and lower-cost (Weichert et al., 2013). Here, the Leap Motion sensor is applied to collect 3D vectors of the human hand.

Two healthy participants aged 22–35 years contributed to a dataset of CSL gestures. They are asked to repeat each gesture 50 times comfortably. The length of each gesture is recorded within 5 s to avoid muscle fatigue and affect data quality. During the data recording, participants are asked to take a break for each repeat. They told the details of data collection in advance. Four different gestures of the right hand are recorded at a frequency of 50 Hz. The LMC data are recorded by the deep cameras located on the sensor facing the participants’ hand. It is worth noting that both participants placed their palms at the same height above the LMC sensor. Also, the positions of the Myo armband for them are the same.

**Figure 4:**
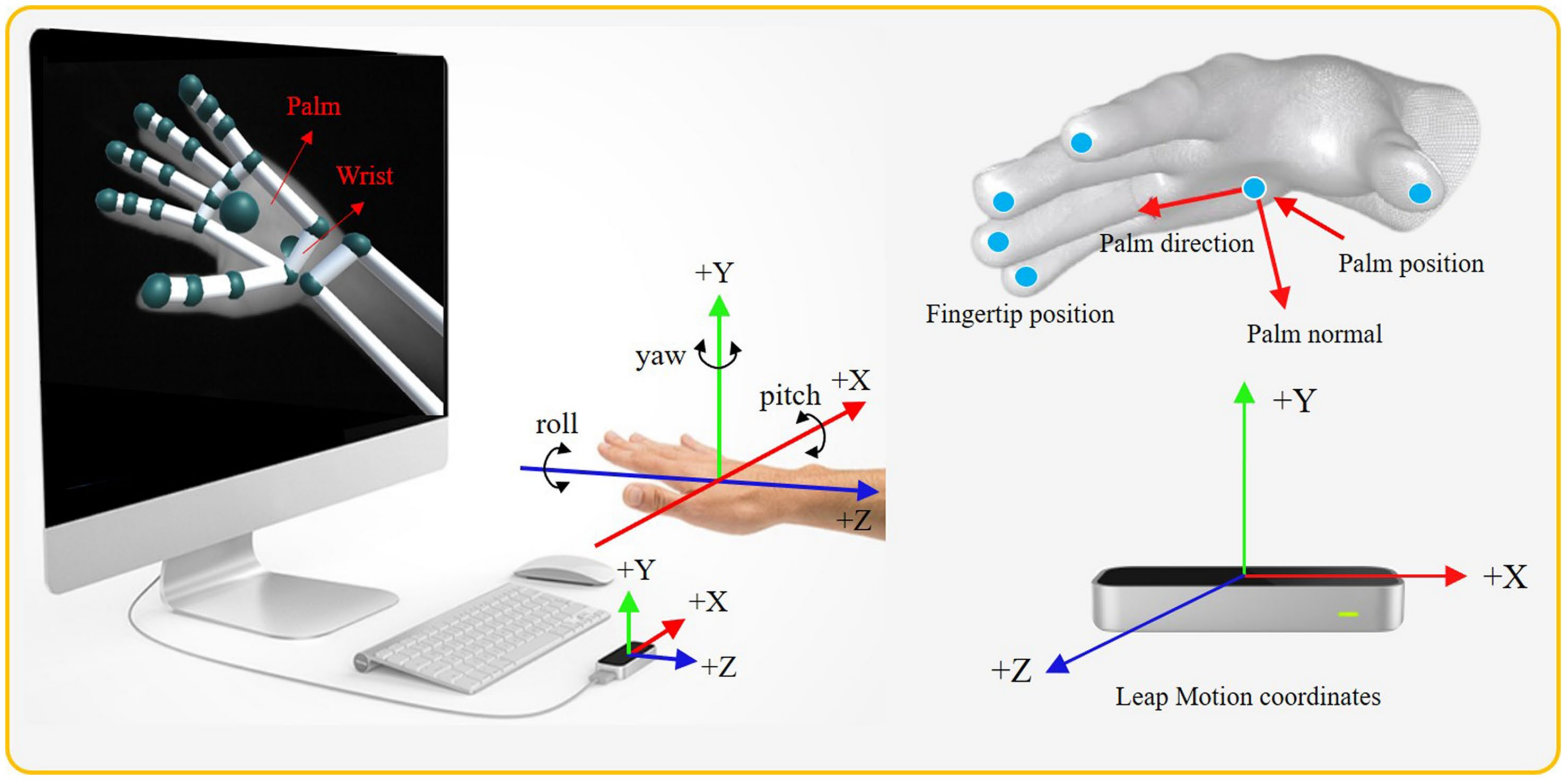
The coordinate system of the Leap Motion sensor and diagram of the bone data detected by it.

Figure 4 demonstrates the fingertips, wrist, and palm position. For each performed gesture, we record all the 3D coordinates of human hand. Then, the start palm position, the difference between the start palm positions, changes of palm positions, palm direction, and velocity of the palm are extracted from these 3D coordinates. As shown in Figure 4, we also extracted the yaw, pitch, and roll of the palms. It is noted that yaw is the angle between the negative z-axis and the projection of the vector onto the x*-*z plane. Similarly, pitch and roll are the angles between the corresponding negative coordinate axes and the projection of corresponding vectors. In other words, pitch, yaw, and roll represent the rotations around the x, y, and z axes, respectively. The angle is calculated through two 3D vectors (Bird et al., 2020). Assuming that the angle θ is constructed by the vectors of $\vec{a}$ and $\vec{b}$, then it can be computed as follows


(1) $$\theta =\arccos\left(\frac{\vec{a}\vec{b}}{\left|\vec{a}\right|\left|\vec{b}\right|}\right)$$

where a and b are vectors made up of two points in space following the LMC coordinate system. The LMC sensor adopts a Cartesian coordinate system based on right-hand. The origin is at the top center of LMC. $\left|\vec{a}\right|$ and $\left|\vec{b}\right|$ are the magnitudes of the corresponding vectors, which can be computed as follows


(2) $$\begin{array}{c} \left|\vec{a}\right|=\sqrt{{\left(a_{x}\right)}^{2}+{\left(a_{y}\right)}^{2}+{\left(a_{z}\right)}^{2}}\;\\ \;\left|\vec{b}\right|=\sqrt{{\left(b_{x}\right)}^{2}+{\left(b_{y}\right)}^{2}+{\left(b_{z}\right)}^{2}\;}\; \end{array}$$

where the subscripts of a and b correspond to the x, y, and z coordinates of each vector in space, respectively.

In this work, nine features (six 3D coordinates and three one-dimensional angle) are chosen to distinguish four CSL gestures. Each 3D coordinate is three-dimensional. Hence, the total dimensions of the nine features are 21, as shown in Table 1. It is known that the features of dynamic gestures are time-varying. The change of palm position can reflect that change well. Hence, the palm position (as shown in the second feature in Table 1) is extracted as one of the features to distinguish dynamic and static gestures effectively. Thus, the proposed framework can recognize both static and dynamic gestures without another special classifier. Noting that the wrist position is extracted to reflect the change of arm.

**Table 1:** Descriptions of CSL collected from leap motion sensor.

Features	Descriptions
Palm position	3D coordinates (X, Y, and Z)
Change of palm position	3D coordinates (X, Y, and Z) The difference between the start and the end position of palm.
Palm normal	3D coordinates (X, Y, and Z)
Palm direction	3D coordinates (X, Y, and Z)
Palm velocity	3D coordinates (X, Y, and Z)
Yaw of the palm	Angle (one dimension)
Pitch of the palm	Angle (one dimension)
Roll of the palm	Angle (one dimension)
Wrist position	3D coordinates (X, Y, and Z)

#### 2.2.2. Human arm sEMG signals captured by Myo armband sensor

**Figure 5:**
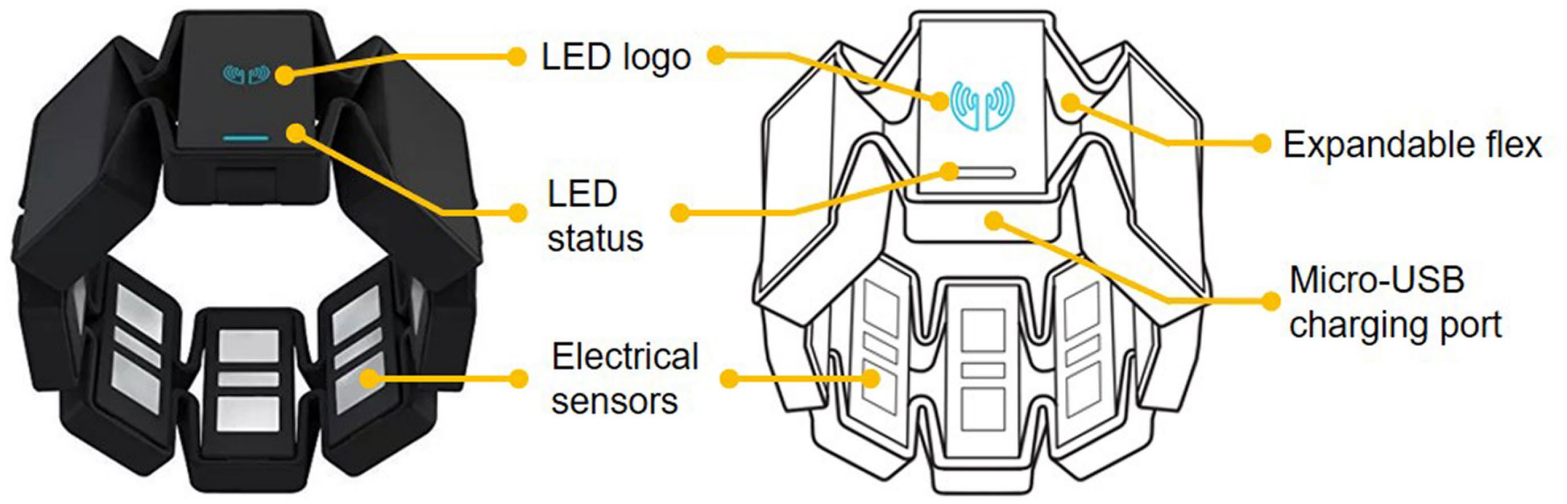
The view of Myo armband.

Figure 5 shows the sketch of Myo armband. It is a wearable and lightweight elastic armband. Myo armband is produced by the Thalmic Labs which consists of several metal contacts. These metal contacts can measure the electrical activity of the user’s forearm muscles. Thus, the Myo armband can recognize their hand gestures and detect their arm motion by reading the electrical activity of human muscles. The Myo armband has eight detection channels. Correspondingly, eight-channel sEMG signals of the human forearm arm are captured to classify sign language gestures together with LMC data. Since gestures are collected synchronously from the Myo armband and Leap Motion sensor, the sampling frequencies for both sensors are the same. The raw sEMG signals are noisy. Therefore, it is necessary to process the signals captured by the Myo armband to train the gesture classifier effectively (Zardoshti-Kermani et al., 1995; Phinyomark et al., 2013; Camargo and Young, 2019).

#### 2.2.3. Data preprocessing for two data subjects

**Figure 6:**
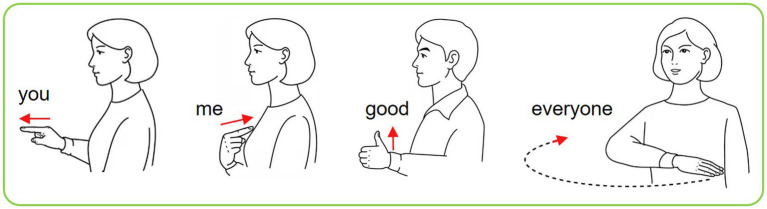
Four kinds of CSL gestures.

Based on the above-mentioned, four CSL gestures recorded from two sensors are depicted in Figure 6. These four gestures are chosen because they are commonly used by Chinese people. The useful right-hand gestures for general conversation include “you,” “me,” “everyone,” and “good.” For the four gestures, only “everyone” is the dynamic gesture.

Before the datasets are fed into the classifier, we must preprocess them to obtain a better recognition result. For the LMC data, each feature is normalized to a value between 0 and 1. The purpose of normalization is to make the preprocessed data limited to a certain range, so as to eliminate the adverse effects (such as causing the training time to increase, which also may lead to the failure of convergence) caused by the singular samples.

As for the Myo data, preprocessing and feature extracting are necessary before training a classifier. Since the sEMG signals are noisy and different features influence the recognition performance, the preprocessing technique is an efficient way to reduce the impact on recognition results caused by the above factors. Low-pass filtering and band-pass filtering are used to preprocess the sEMG signals first. The low-pass filtering is aimed at obtaining signals with a frequency of 5–200 Hz, and band-pass filtering is used to obtain the envelope of sEMG signals. Then, the root mean square (RMS; Kundu et al., 2018; Le Sant et al., 2019) is extracted as a feature of sEMG signals. Compared with other features, such as waveform length (Phinyomark et al., 2009; Arief et al., 2015), and autoregressive model features (Subasi, 2012; Krishnan et al., 2019), it has been verified that the RMS feature obtain the best result under different lengths of sampling moving window (Luo et al., 2020).

#### 2.2.4. Data fusion of two modalities data

After preprocessing, we can obtain two datasets from LMC and Myo armband sensors. Recent studies have shown that sensor fusion can promote richness, completeness, and accuracy of information with less uncertainty to enhance the performance of training (Chavez-Garcia and Aycard, 2015; Li et al., 2020). Here, feature-level fusion is applied to fuse information of two sensors. Two preprocessed sequences are merged into a longer sequence with 29 dimensions as input of the LSTM network. In other words, each gesture has 29 features. For each gesture, the data collected from both sensors have a history of 50 frames. Thus, the size of each gesture is 50*29.

### 2.3. Deep learning classification approaches

Recurrent neural network (RNN) is a commonly used approach in training and classifying time-series data. However, it is easy to occur gradients explosion or vanish when RNN handles long-term dependence. LSTM is designed to solve this problem. Compared with general RNN, LSTM performs better in learning longer time-series data. In this work, the LSTM network is used to classify the multimodal CSL gesture sequences.

**Figure 7:**
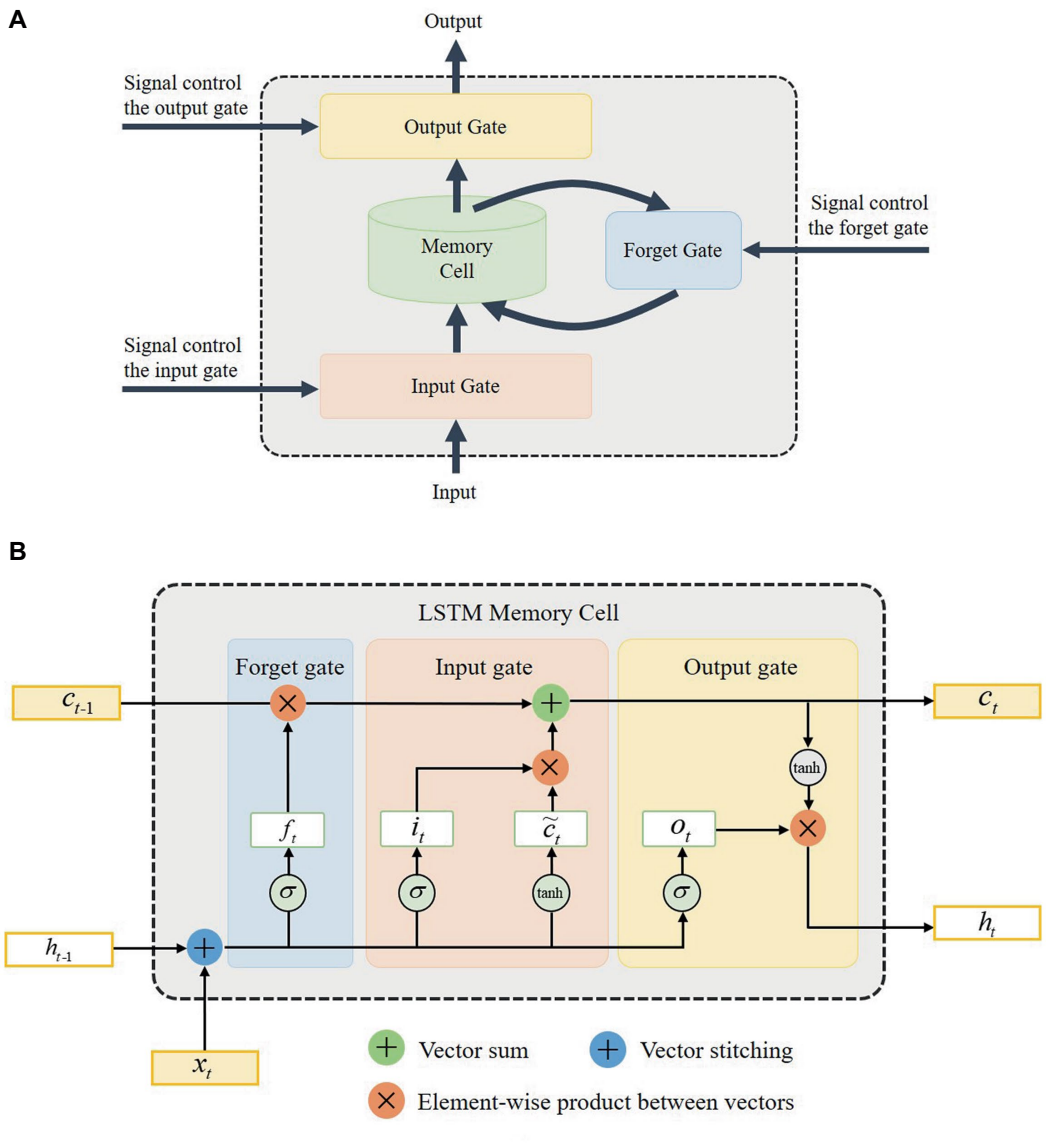
The architecture of the long short-term memory (LSTM) network. **(A)** The composition of LSTM memory blocks. **(B)** The structure of LSTM memory cell.

The key to the effectiveness of LSTM in dealing with sequence problems lies in memory blocks and gates (Hochreiter and Schmidhuber, 1997). As shown in Figure 7A, each memory block consists of an input gate, a memory cell, an output gate, and a forget gate. The memory cell retains information relying on different time intervals. The input gate, forget gate, and output gate determine whether the information flow can enter or exit the memory cell. Three independent gates work together to ensure that the cell retains information for a long time. Figure 7B shows the actual structure of the LSTM memory cell.

As shown in Figure 7B, $x_{t}$ is the input of the LSTM network, and $h_{t}$ is the output of the network. $f_{t}$, $i_{t}$, and $o_{t}$ respectively denote the forget gate, input gate, and output gate variables of the LSTM network. The subscripts t and t−1 represent the current time and previous time. $c_{t}$ is the memory cell state. The notation of σ and tanh denote sigmoid and hyperbolic activation functions, respectively. With the memory gates, the input, output, and key parameters of the LSTM network can be computed (Graves, 2013)


(3) $$i_{t}=\sigma \left(W_{ix}x_{t}+W_{ih}h_{t-1}+W_{ic}c_{t-1}+b_{i}\right)$$

(4) $$f_{t}=\sigma \left(W_{fx}x_{t}+W_{fh}h_{t-1}+W_{fc}c_{t-1}+b_{f}\right)$$

(5) $$c_{t}=f_{t}c_{t-1}+i_{t}\tanh\left(W_{cx}x_{t}+W_{ch}h_{t-1}+b_{c}\right)$$

(6) $$o_{t}=\sigma \left(W_{ox}x_{t}+W_{oh}h_{t-1}+W_{oc}c_{t}+b_{o}\right)$$

(7) $$h_{t}=o_{t}\tanh\left(c_{t}\right)$$

where subscripts i, o, f, and c respectively represent the parameters related to the input gate, output gate, forget gate, and memory cell. The subscripts of the weight matrix are similar. For instance, $W_{ih}$ denotes the input-hidden matrix, $W_{ic}$ denotes the input-memory cell matrix, etc. Similarly, $b_{f}$, $b_{i}$, $b_{c}$, and $b_{o}$ present the biases of corresponding subscripts for the LSTM network. tanh is the hyperbolic activation function, while σ is the sigmoid activation function.

The special structure of the memory cell endows the LSTM network with powerful capability in modeling time-based sequences with long-range dependencies. Therefore, the applications of this network have covered a great many fields successfully. In this work, the LSTM network is used to classify the time-series CSL gestures. With this network, the CSL gestures can be classified well. Then, the classification results will be sent to a social robot for interaction and reaction.

This section first outlined the proposed framework and briefly introduced each module of this framework. Then, the collection of the sign language datasets, and the preprocessing and fusion of two different sensor data were elaborated in detail. Lastly, the relevant classification algorithm makes the above datasets suitable to our framework was presented.

## 3. Experiments and results

Two experiments were performed to verify the proposed HRI framework. First, we compare the recognition performance of sensor fusion-based multimodal gesture datasets with individual sensor datasets. Then, we test the proposed framework according to several gesture recognition results and reactions with the NAO robot using LMC and Myo armbands.

### 3.1. Experimental setup

The experimental platform is introduced below:

#### 3.1.1. Hardware platform

The experimental devices mainly include two gesture collection sensors and a social robot. As aforementioned, the Myo armband and LMC sensors are used to collect eight-channel sEMG signals and hand 3D information, respectively. The social robot applied in the experiment is a NAO robot. As a bipedal humanoid robot, NAO is produced by the French Aldebaran Robotics Company. It is currently the most influential social robot research platform (Bartneck et al., 2019). Because the robot is low cost, easy to program, small in size, portable, and able to conduct research outside the laboratory (Su et al., 2007; Cohen et al., 2011; Garimort et al., 2011). Therefore, it has become a widely used robotic platform for HRI research by academic institutions around the world. Here, it is used to communicate with a person by gestures.

#### 3.1.2. Software environment

The LSTM classifier was run on an Intel i7-4600M CPU with 2.9GHZ which has 8 GB of GDDR5 memory. The LSTM model was built using the Python 3.6 library of Keras and trained using fusion data. Control software of NAO robot Choregraphe is employed to interact *via* gestures with a specified person. Both software runs on Windows 10 operating system.

### 3.2. Multimodal gestures comparison experiments and results

The demonstration data from the Kinect sensor and Myo armband will be preprocessed before it is fed into the incremental learning method. Firstly, the data fusion method based on the KF is used to fuse the joint angles and joint angular velocities to obtain a more accurate and smooth dataset. Since the demonstration data are not matched in the timeline, then the dynamic time warping (DTW) algorithm is applied to align it. Here, the two preprocessing methods will be introduced briefly.

#### 3.2.1. Settings

The first experiment is performed to test the recognition performance for multimodal gestures. To compare with single modality data, three different sensor datasets are fed into the LSTM classifier. The corresponding conditions are considered as follows.

Condition 1: Single modality data from LMC sensor. The input data of the LSTM network are the 21-dimension (as listed in Table 1) 3D hand vectors collected from the LMC sensor.
Condition 2: Single modality data from Myo armband. In other word, the input data of the LSTM network are the eight-channel sEMG signals of the human forearm arm with eight dimensions.
Condition 3: Two modality sensors data from two sensors (Leap Motion sensor and Myo armband). In this condition, the input data of the LSTM network is the combination of the 21-dimension 3D hand vectors and the eight-dimension sEMG signals of the human forearm arm. Before the data are fed into the network, the two sensors datasets are preprocessed and normalized, respectively. Then, the normalized datasets are fused as a new input vector of the LSTM.

In the conditions 1 and 2, the steps are the same except that the input data is different. The LSTM model is trained by feeding each of the time-series training data in batches of 10. And this is performed over 100 epochs of training. There are 400 sequences for four CSL gestures in total. The data set is randomly divided into training data and cross-validation data at a ratio of 90–10, respectively. It means that the number of training and testing sets is 360 and 40, respectively.

**Figure 8:**
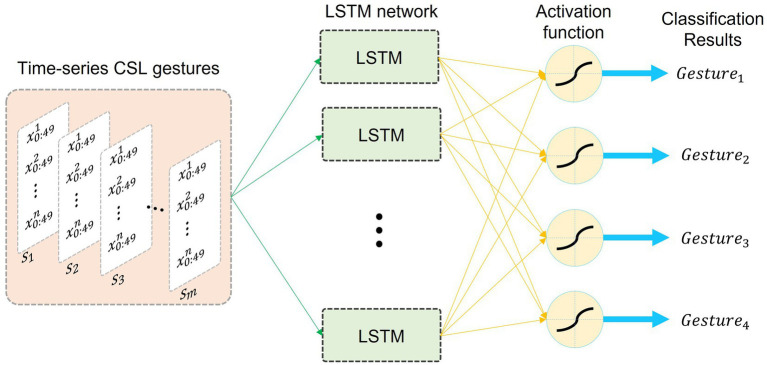
The LSTM model used in the experiment.

There are two important parameters for the LSTM network that can improve the classification results. One is the number of hidden neurons, and the other is the epoch. To obtain the optimal performance, the number of hidden neurons and epochs for the LSTM network under the above conditions are successively valued from 1 to 150. Table 2 shows the parameters setup of the LSTM network under three conditions. Figure 8 shows the model of the LSTM network. The superscript n of $x_{0:49}^{n}$ denotes the dimensions of gesture features. The values of n are different under the above three conditions. The subscript of $x_{0:49}^{n}$ is the length of each gesture sample. The subscript m of $S_{m}$ denotes the number of gesture samples.

**Table 2:** Parameters setup for the first three experiments.

Parameters	Condition 1	Condition 2	Condition 3
Size of input	50∗21	50*8	50*29
Size of output	4	4	4
Number of hidden neurons	20	20	15
Epoch	50	50	30
Batch size	10	10	10

For each epoch, the training and test accuracy are computed and echoed. The computation of accuracy is as follows


(8) $$\mathrm{Accuracy}=\frac{Ges_{\mathrm{correct}}}{Ges_{\mathrm{total}}}$$

where $Ges_{\mathrm{correct}}$ denotes the number of gestures classified correctly. $Ges_{\mathrm{total}}$ represents the total number of gesture samples collected.

#### 3.2.2. Results and analysis

**Figure 9:**
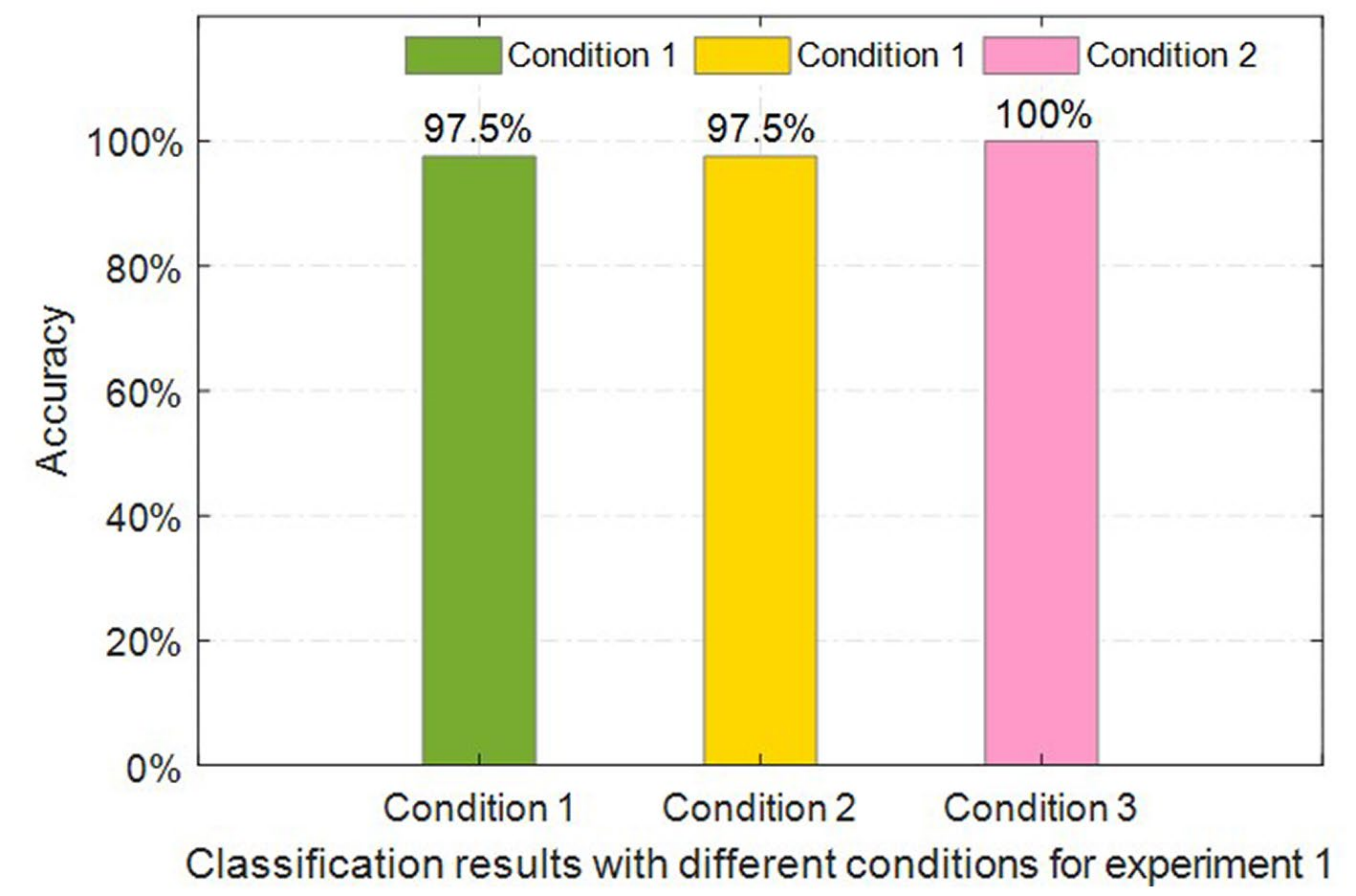
Classification accuracies under three conditions for the first experiment.

For all conditions, the training processes were performed and repeated several times to obtain a better model of the LSTM classifier. At the end of all the epochs of training, the model is made to test with the cross-validation data and its accuracy is also echoed. To prevent overfitting, the model is trained over 100 times. At each time, the loss and accuracy are noted. At the end of each training, the model is saved. The model with the least loss and highest cross-validation accuracy is chosen for use in the second experiment. The classification results of CSL gestures under three conditions are shown in Figure 9.

Obviously, the classification accuracy under condition 3 achieves 100%, while the accuracy could not achieve that under conditions 1 and 2. In other words, the multimodal sensor fusion-based input data obtains a better performance in comparison with that of single-modality sensor data. The recognition accuracies under conditions 2 and 3 are the same when the single modality sensor datasets are used.

**Figure 10:**
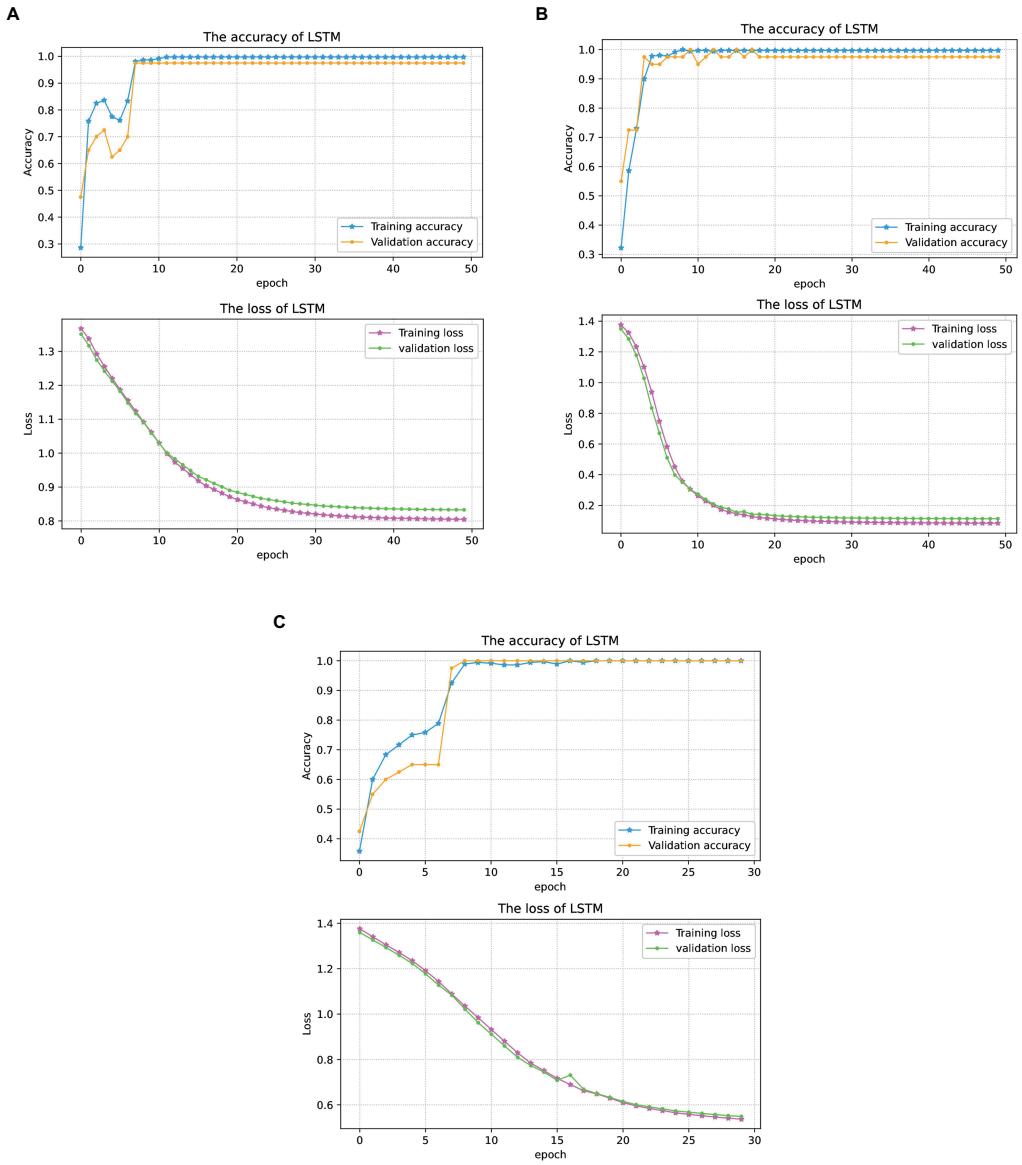
The first experiment results under three conditions. **(A)** CSL gestures classification results under condition 1. **(B)** CSL gestures classification results under condition 2. **(C)** CSL gestures classification results under condition 3. In panels **(A–C)**, the blue curves with star markers denote the recognition accuracy of the training set, and the yellow curves with circle markers denote the recognition accuracy of the testing set. The magenta and green curves are the loss values of the training sets and testing sets, respectively.

Figure 10 shows the classification results corresponding to three conditions of the above-mentioned scenarios. With the increase of training epochs, loss gradually converges to three different values corresponding to three conditions. It means that the multimodal fusion data achieves the highest recognition accuracy with convergent loss values.

**Figure 11:**
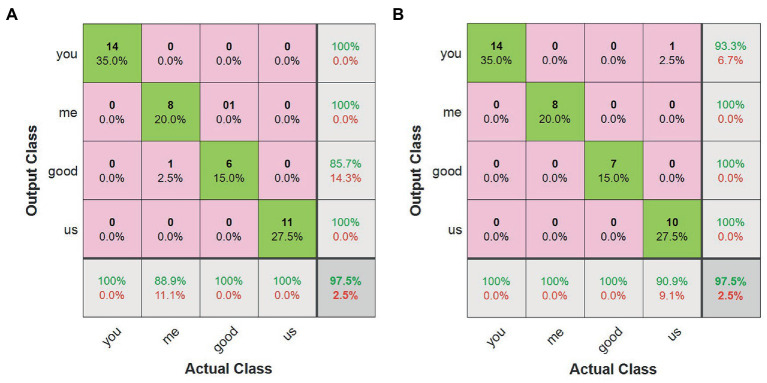
The confusion matrices of the first experiment under conditions 1 and 2. **(A)** The confusion matrix under condition 1. **(B)** The confusion matrix under condition 2. In panels **(A,B)**, *x*-axis denotes the real sample labels, and the *y*-axis denotes the predicted sample labels. The top and bottom elements on the main diagonal filled with green color, respectively, represent the number and percentage of the samples that are correctly predicted. The top and bottom elements inside of each pink square, respectively, represent the number and percentage of wrong predicted samples. The top and bottom elements inside of lower and right light gray squares represent the prediction accuracy and error rate of corresponding samples.

As shown in Figure 11, the confusion matrices under three conditions are presented to explore the impact on classification results based on misclassified gestures and different modality data. The recognition accuracy under condition 3 is 100%, which means that all testing gestures are correctly recognized. Hence, we will not discuss the confusion matrix under condition 3. From Figure 11, we can find that only one CSL gesture is classified incorrectly under conditions 1 and 2 in the test samples. This is because both of the conditions have the same recognition accuracy. But the misclassified gestures are not the same. The misclassified gesture type is “you” under condition 1, and that is “me” under condition 2. That is probably because the two gestures have the same postures except for directions.

### 3.3. HRI experiments and results

#### 3.3.1. Settings

**Figure 12:**
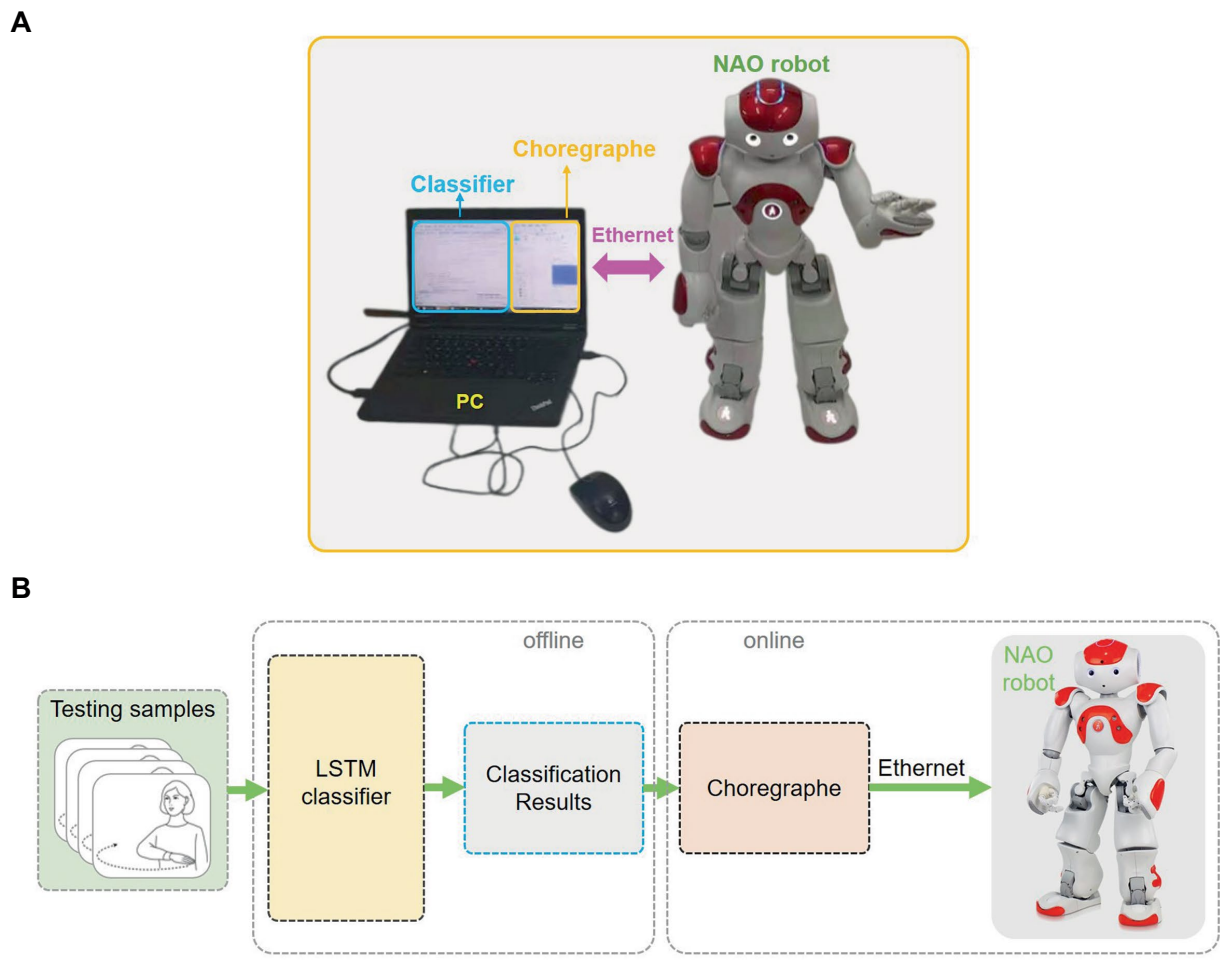
The experimental system of the second experiment. **(A)** The experimental platform of the second experiment. **(B)** The experimental steps of the second experiment.

The second experiments were conducted on an NAO robot based on the first experiments. Firstly, two different modalities of testing CSL gestures were sent to the LSTM classifier. Then, the recognition results were transported to the NAO robot for understanding and reaction. Based on the recognition results, Choregraphe APP converts the corresponding gestures into executable commands so that the robot can perform and respond. In other words, the output of the classifiers being coded into commands for the robot’s response. Hence, the recognition of human hand motion for the robot is from the system. Choregraphe connects robots via Ethernet. The experimental platform and experiment steps are shown in Figure 12. Once these gestures are classified and sent to Choregraphe, the corresponding responses of the NAO robot will be performed.

Noting that the classifier model under the third condition in the first experiment is saved to recognize the testing gestures in this experiment. Testing data include two types of gestures: four kinds of captured singular gestures and two combination gestures consisting of them. The combined CSL gesture is composed according to Chinese grammar which can express a complete meaning. The testing gestures are shown in Table 3. In Chinese, “hello” is a combination of the two words “you” and “good,” and “hello, everyone” is a combination of the three words “you,” “us,” and “good.” As shown in Table 3, six gestures are tested in total for the second experiment.

**Table 3:** All testing gestures in the second experiment.

Type	Gestures
Four singular hand gestures	Good
	You
	Us
	Me
Two combination gestures	Hello (combination of you and Good gestures)
	Hello, everyone (combination of you, us, and Good gestures)

#### 3.3.2. Results and analysis

**Figure 13:**
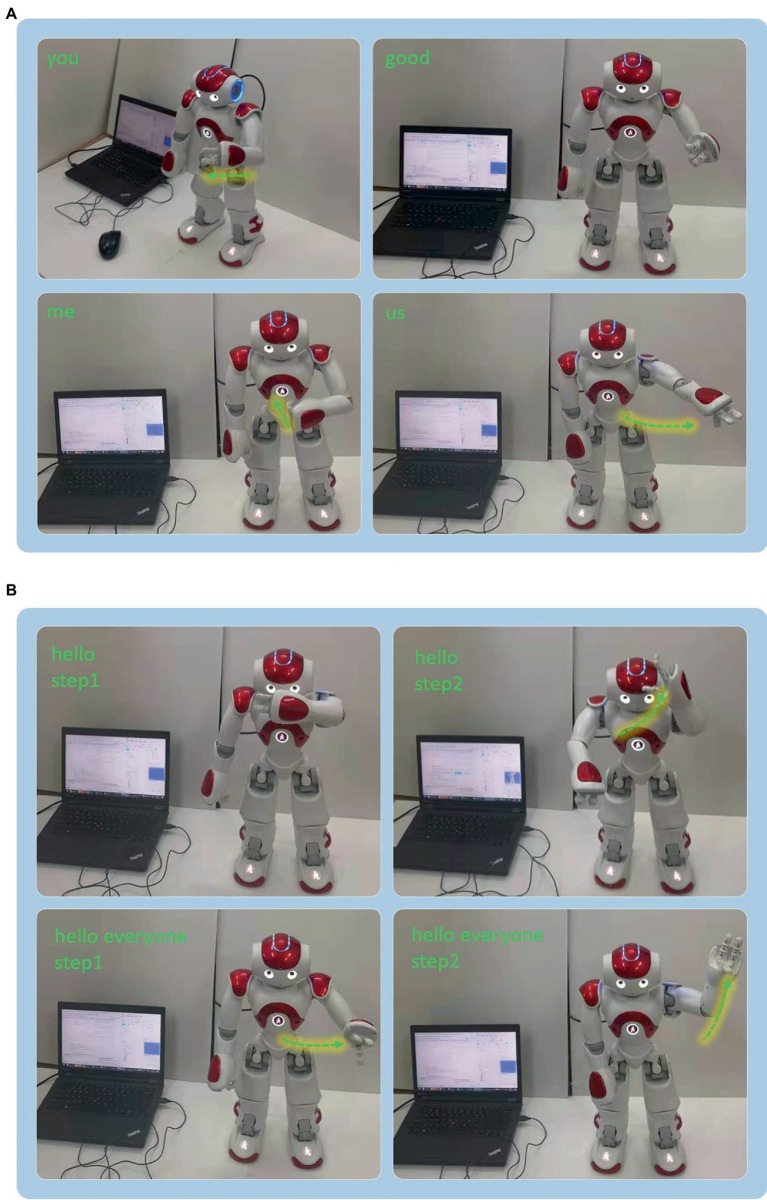
The NAO robot interaction results of experiment 2. **(A)** Robot response results of the four singular hand gestures. **(B)** Robot response results of the two combination gestures.

The experiment was performed more than 10 times for each gesture. Figure 13 shows the response results of the NAO robot corresponding to the six gestures. In Figure 13A, the words in the upper right corner are four kinds of gesture results recognized by the NAO robot and the gestures of NAO are the corresponding response results. In Figure 13B, the response results of the NAO robot gesture are divided into two steps for each combination gesture. Obviously, the robot’s responses to the six gestures are different. For the single gestures, the robot’s response is only one step. However, the response according to the combination gestures is two steps. This implies that the proposed framework can interact with people through CSL gestures and react with reasonable responses. It also indicated that the proposed system can not only interact with the robot based on a single gesture but also interact through a combination of gestures.

## 4. Discussion

Two experiments are conducted to verify the effectiveness of the proposed framework. According to the first experimental results, we can conclude that the multimodal sensor data can effectively improve recognition accuracy, similar to the experimental findings of Zeng et al. (2019) and Zeng et al. (2020). The confusion matrices of experiment 1 under conditions 1 and 2 imply that different single-modal sensor data can classify different kinds of gestures. Leap Motion sensor data can achieve a good result in human hand posture by capturing a 3D skeletal hand model. The Myo armband sensor can obtain better results in gestures with significant differences in sEMG signals. This also demonstrates that different modal sensor data provides complementary information. Hence, the fused multimodal data achieves the best results in the first experiment.

To investigate the application of our proposed framework in HRI and its advantages in CSL gesture classification, we performed another experiment. In general, most of the conventional gesture classification frameworks can only classify singular static or dynamic sign language gestures. However, our SLR framework can classify both singular and combination gestures well. This combination is not only in terms of gestures but also in terms of the special framework. As the input of the LSTM network, the dynamic and static gestures samples are mixed in one dataset. We can distinguish them effectively by the specific features captured from the LMC. The second experimental results have proved that. In addition, our proposed SLR framework also can be applied in other HRI scenarios. It provides a novel way for the SLR application in social robots and provides a compatible SLR framework.

## 5. Conclusion and future work

This paper presented a multimodal CSL recognition framework applied in HRI between deaf-mutes and social robots. The multimodal framework considers multiple sensor information for the human hand and arm, including human 3D vector and arm sEMG signals. The Leap Motion sensor and Myo armband are used to capture corresponding signals. Then, the preprocessing techniques are carried out aimed at reducing the training process to improve recognition accuracy to some extent. For LMC data, the normalization method is to limit data to a certain range to eliminate the adverse effects of singular samples. Since the sEMG signals are noisy, low-pass filtering and band-pass filtering are used to preprocess the signals. After that, the RMS feature is extracted from sEMG signals and fused with Leap Motion data as the input data of the classifier. Our method fuses the sensor data from a wearable and vision-based devices at the feature level. Comparative experiments have validated the method. The proposed multimodal framework can facilitate deaf and speech-impaired people to learn sign language through a social robot with the ability of SLR. Our future work will concentrate on developing a framework with a stronger generalization capability to recognize various sign languages without the limitation of country and language restrictions.

## Data availability statement

The raw data supporting the conclusions of this article will be made available by the authors, without undue reservation.

## Author contributions

JL: human-robot interaction system design, methodology, and experiments. JL and NW: results analysis. JL: manuscript writing and original draft. JL, JZ, and NW: review and editing. JL and JZ: funding acquisition. Each author has read and edited the manuscript, and agrees with its content. All authors contributed to the article and approved the submitted version.

## Funding

This work was supported by the Startup Foundation of Chongqing Technology and Business University under Grant No. 950321049 and No. 2056019, and partially supported by the Germany/Hong Kong Joint Research Scheme sponsored by the Research Grants Council of Hong Kong and the German Academic Exchange Service of Germany (Ref. No. G-PolyU505/22), PolyU Start-up Grant: ZVUY-P0035417, CD5E-P0043422 and WZ09-P0043123.

## Conflict of interest

The authors declare that the research was conducted in the absence of any commercial or financial relationships that could be construed as a potential conflict of interest.

## Publisher’s note

All claims expressed in this article are solely those of the authors and do not necessarily represent those of their affiliated organizations, or those of the publisher, the editors and the reviewers. Any product that may be evaluated in this article, or claim that may be made by its manufacturer, is not guaranteed or endorsed by the publisher.

## Footnotes

^1^World Federation of the Deaf. (2023). E. coli. http://wfdeaf.org/our-work/. [Accessed February 8, 2023].

## References

[ref1] AI Farid F. Hashim N. Abdullah J. Bhuiyan M. R. Shahida Mohd Isa W. N. Uddin J.. (2022). A structured and methodological review on vision-based hand gesture recognition system. J. Imag. 8:153. doi: 10.3390/jimaging8060153, PMID: 35735952 PMC9224857

[ref2] Arief Z. Sulistijono I. A. Ardiansyah R. A. (2015). “Comparison of five time series EMG features extractions using Myo armband.” in *The 2015 international electronics symposium (Surabaya)*. 11–14.

[ref3] Bartneck C. Belpaeme T. Eyssel F. Kanda T. Keijsers M. Sabanovic S. (2019). Human robot interaction. Hum. Robot Interact Introduct., 6–17. Available at: https://sc.panda321.com/#v=onepage&q=Human%20robot%20interaction&f=false

[ref4] Bird J. J. Ekárt A. Faria D. R. (2020). British sign language recognition via late fusion of computer vision and leap motion with transfer learning to american sign language. Sensors 20:5151. doi: 10.3390/s20185151, PMID: 32917024 PMC7571093

[ref5] Breazeal C. Dautenhahn K. Kanda T. (2016). “Social robotics” in Springer Handbook of Robotics, 1935–1972. Available at: https://link.springer.com/chapter/10.1007/978-3-319-32552-1_72

[ref6] Camargo J. Young A. (2019). Feature selection and non-linear classifiers: effects on simultaneous motion recognition in upper limb. IEEE Trans. Neural Syst. Rehabil. Eng. 27, 743–750. doi: 10.1109/tnsre.2019.2903986, PMID: 30869626

[ref7] Camgoz N. C. Hadfield S. Koller O. Ney H. Bowden R. (2018). “Neural sign language translation” in the *IEEE Conference on Computer Vision and Pattern Recognition (Utah)*. 7784–7793.

[ref001] Cao H. Chen G. Li Z. Feng Q. Lin J. Knoll A. (2022a). Efficient Grasp Detection Network With Gaussian-Based Grasp Representation for Robotic Manipulation. IEEE/ASME Transactions on Mechatronics. doi: 10.1109/TMECH.2022.3224314

[ref002] Cao H. Chen G. Li Z. Hu Y. Knoll A. (2022b). NeuroGrasp: multimodal neural network with Euler region regression for neuromorphic vision-based grasp pose estimation. IEEE Transactions on Instrumentation and Measurement 71, 1–11. doi: 10.1109/TIM.2022.3179469

[ref8] Chavez-Garcia R. O. Aycard O. (2015). Multiple sensor fusion and classification for moving object detection and tracking. IEEE Trans. Intell. Transp. Syst. 17, 525–534. doi: 10.1109/tits.2015.2479925

[ref003] Chen G. Cao H. Conradt J. Tang H. Rohrbein F. Knoll A. (2020). Event-based neuromorphic vision for autonomous driving: A paradigm shift for bio-inspired visual sensing and perception. IEEE Signal Processing Magazine. 37, 34–49. doi: 10.1109/MSP.2020.2985815

[ref9] Cheok M. J. Omar Z. Jaward M. H. (2019). A review of hand gesture and sign language recognition techniques. Int. J. Mach. Learn. Cybern. 10, 131–153. doi: 10.1007/s13042-017-0705-5

[ref10] Chong T. W. Lee B. G. (2018). American sign language recognition using leap motion controller with machine learning approach. Sensors 18:3554. doi: 10.3390/s18103554, PMID: 30347776 PMC6210690

[ref11] Cohen I. Looije R. Neerincx M. A. (2011). “Child's recognition of emotions in robot's face and body” in the *6th International Conference on Human-robot Interaction*. 123–124.

[ref12] Cui R. Liu H. Zhang C. (2019). A deep neural framework for continuous sign language recognition by iterative training. IEEE Trans. Multimedia 21, 1880–1891. doi: 10.1109/TMM.2018.2889563

[ref13] Garimort J. Hornung A. Bennewitz M. (2011). “Humanoid navigation with dynamic footstep plans” in the *2011 IEEE International Conference on Robotics and Automation (Shanghai)*. 3982–3987.

[ref14] Gonzalez-Aguirre J. A. Osorio-Oliveros R. Rodríguez-Hernández K. L. Lizárraga-Iturralde J. Morales Menendez R. Ramírez-Mendoza R. A.. (2021). Service robots: trends and technology. Appl. Sci. 11:10702. doi: 10.3390/app112210702

[ref15] Graves A. (2013). Generating sequences with recurrent neural networks. arXiv [Preprint]. doi: 10.48550/arXiv.1308.0850

[ref16] Hochreiter S. Schmidhuber J. (1997). Long short-term memory. Neural Comput. 9, 1735–1780. doi: 10.1162/neco.1997.9.8.1735 9377276

[ref17] Hong P. Turk M. Huang T. S. (2000). “Gesture modeling and recognition using finite state machines.” in *The 4th IEEE International Conference on Automatic Face and Gesture Recognition (Grenoble)*. 410–415

[ref18] Karami A. Zanj B. Sarkaleh A. K. (2011). Persian sign language (PSL) recognition using wavelet transform and neural networks. Expert Syst. Appl. 38, 2661–2667. doi: 10.1016/j.eswa.2010.08.056

[ref19] Krishnan S. Akash R. Kumar D. Jain R. Rathai K. M. M. Patil S. (2019). “Finger movement pattern recognition from surface EMG signals using machine learning algorithms.” in the 2017 International Conference on Translational Medicine and Imaging (Vellore). 2017, 75–89.

[ref20] Kumar P. Gauba H. Roy P. P. Dogra D. P. (2017a). A multimodal framework for sensor based sign language recognition. Neurocomputing 259, 21–38. doi: 10.1016/j.neucom.2016.08.132

[ref21] Kumar P. Gauba H. Roy P. P. Dogra D. P. (2017b). Coupled HMM-based multi-sensor data fusion for sign language recognition. Pattern Recogn. Lett. 86, 1–8. doi: 10.1016/j.patrec.2016.12.004

[ref22] Kundu A. S. Mazumder O. Lenka P. K. Bhaumik S. (2018). Hand gesture recognition based omnidirectional wheelchair control using IMU and EMG sensors. J. Intell. Robot. Syst. 91, 529–541. doi: 10.1007/s10846-017-0725-0

[ref23] Kurdyumov R. Ho P. Ng J. (2011). Sign language classification using webcam images. Comput. Therm. Sci. 10:9029. Available at: http://cs229.stanford.edu/proj2011/KurdyumovHoNg-SignLanguageClassificationUsingWebcamImages.pdf

[ref24] Le Sant G. Gross R. Hug F. Nordez A. (2019). Influence of low muscle activation levels on the ankle torque and muscle shear modulus during plantar flexor stretching. J. Biomech. 93, 111–117. doi: 10.1016/j.jbiomech.2019.06.018, PMID: 31280899

[ref25] Li J. Zhong J. Chen F. Yang C. (2019). “An incremental learning framework for skeletal-based hand gesture recognition with leap motion.” In The IEEE 9th Annual International Conference on CYBER Technology in Automation. Control, and Intelligent Systems (Suzhou). 13–18.

[ref26] Li J. Zhong J. Yang J. Yang C. (2020). An incremental learning framework to enhance teaching by demonstration based on multimodal sensor fusion. Front. Neurorobot. 14:55. doi: 10.3389/fnbot.2020.00055, PMID: 32982712 PMC7481388

[ref27] Lichtenauer J. F. Hendriks E. A. Reinders M. J. (2008). Sign language recognition by combining statistical DTW and independent classification. IEEE Trans. Pattern Anal. Mach. Intell. 30, 2040–2046. doi: 10.1109/TPAMI.2008.123, PMID: 18787250

[ref28] Luo J. Liu C. Feng Y. Yang C. (2020). A method of motion recognition based on electromyographic signals. Adv. Robot. 34, 976–984. doi: 10.1080/01691864.2020.1750480

[ref29] Mitra S. Acharya T. (2007). Gesture recognition: a survey. IEEE Trans. Syst. Man Cyber. C. 37, 311–324. doi: 10.1109/TSMCC.2007.893280

[ref30] Naglot D. Kulkarni M. (2016). “Real time sign language recognition using the leap motion controller” in the *2016 International Conference on Inventive Computation Technologies* (Karnataka). 3, 1–5.

[ref31] Oudah M. Al-Naji A. Chahl J. (2020). Hand gesture recognition based on computer vision: a review of techniques. J. Imag. 6:73. doi: 10.3390/jimaging6080073, PMID: 34460688 PMC8321080

[ref32] Phinyomark A. Limsakul C. Phukpattaranont P. (2009). A novel feature extraction for robust EMG pattern recognition. arXiv [Preprint]. doi: 10.48550/arXiv.0912.3973

[ref33] Phinyomark A. Quaine F. Charbonnier S. Serviere C. Tarpin-Bernard F. Laurillau Y. (2013). EMG feature evaluation for improving myoelectric pattern recognition robustness. Expert Syst. Appl. 40, 4832–4840. doi: 10.1016/j.eswa.2013.02.023

[ref34] Pu J. Zhou W. Li H. (2018). “Dilated convolutional network with iterative optimization for continuous sign language recognition.” in the *27th International Joint Conference on Artificial Intelligence (Stockholm)*. 885–891.

[ref35] Qi W. Ovur S. E. Li Z. Marzullo A. Song R. (2021). Multi-sensor guided hand gesture recognition for a teleoperated robot using a recurrent neural network. IEEE Robot. Automat. Lett. 6, 6039–6045. doi: 10.1109/LRA.2021.3089999

[ref36] Rastgoo R. Kiani K. Escalera S. (2020). Video-based isolated hand sign language recognition using a deep cascaded model. Multimed. Tools Appl. 79, 22965–22987. doi: 10.1007/s11042-020-09048-5

[ref37] Roda-Sanchez L. Garrido-Hidalgo C. García A. S. Olivares T. Fernández-Caballero A. (2023). Comparison of RGB-D and IMU-based gesture recognition for human-robot interaction in remanufacturing. Int. J. Adv. Manuf. Technol. 124, 3099–3111. doi: 10.1007/s00170-021-08125-9

[ref38] Si Y. Chen S. Li M. Li S. Pei Y. Guo X. (2022). Flexible strain sensors for wearable hand gesture recognition: from devices to systems. Adv. Intellig. Syst. 4:2100046. doi: 10.1002/aisy.202100046

[ref39] Siciliano B. Khatib O. (2016). Springer Handbook of Robotics Springer, 1–6. Available at: https://link.springer.com/book/10.1007/978-3-319-32552-1?source=shoppingads&locale=en-jp&gclid=Cj0KCQiAwJWdBhCYARIsAJc4idCcF2us102UDVGHpi9py3j3kDIRfTV8W-cT0Jx8dgDKWGwDZj2053EaAqIdEALw_wcB

[ref40] Su Z. W. Huang C. Q. Pan W. (2007). A study on obstacle avoidance for Nao robot based on Webots platform.

[ref41] Subasi A. (2012). Classification of EMG signals using combined features and soft computing techniques. Appl. Soft Comput. 12, 2188–2198. doi: 10.1016/j.asoc.2012.03.035

[ref42] Tharwat A. Gaber T. Hassanien A. E. Shahin M. K. Refaat B. (2015). Sift-based arabic sign language recognition system. Adv. Intellig. Syst. Comput. 334, 359–370. doi: 10.1007/978-3-319-13572-4_30

[ref43] Wang L. Hu W. Tan T. (2003). Recent developments in human motion analysis. Pattern Recogn. 36, 585–601. doi: 10.1016/S0031-3203(02)00100-0

[ref44] Wei C. Zhou W. Pu J. Li H. (2019). “Deep grammatical multi-classifier for continuous sign language recognition” in *The 5th International Conference on Multimedia Big Data (Singapore)*. 435–442.

[ref45] Weichert F. Bachmann D. Rudak B. Fisseler D. (2013). Analysis of the accuracy and robustness of the leap motion controller. Sensors 13, 6380–6393. doi: 10.3390/s130506380, PMID: 23673678 PMC3690061

[ref46] Wong S. F. Cipolla R. (2005). “Real-time adaptive hand motion recognition using a sparse bayesian classifier.” in the *International Workshop on Human-Computer Interaction*. 170–179.

[ref47] Wu Y. Huang T. S. (1999). “Vision-based gesture recognition: a review.” in International Gesture Workshop (France). 103–115.

[ref48] Wu D. Pigou L. Kindermans P. J. Le N. D. H. Shao L. Dambre J.. (2016). Deep dynamic neural networks for multimodal gesture segmentation and recognition. IEEE Trans. Pattern Anal. Mach. Intell. 38, 1583–1597. doi: 10.1109/tpami.2016.2537340, PMID: 26955020

[ref49] Xue Y. Ju Z. Xiang K. Chen J. Liu H. (2018). Multimodal human hand motion sensing and analysis—a review. IEEE Trans. Cogn. Dev. Syst. 11, 162–175. doi: 10.1109/tcds.2018.2800167

[ref50] Yang C. Chen C. He W. Cui R. Li Z. (2018a). Robot learning system based on adaptive neural control and dynamic movement primitives. IEEE Trans. Neural Net. Learn. Syst. 30, 777–787. doi: 10.1109/TNNLS.2018.2852711, PMID: 30047914

[ref51] Yang C. Chen C. Wang N. Ju Z. Fu J. Wang M. (2018b). Biologically inspired motion modeling and neural control for robot learning from demonstrations. IEEE Trans. Cogn. Dev. Syst. 11, 281–291. doi: 10.1109/TCDS.2018.2866477

[ref52] Ye Y. Tian Y. Huenerfauth M. Liu J. (2018). “Recognizing american sign language gestures from within continuous videos” in *The IEEE Conference on Computer Vision and Pattern Recognition Workshops*. 2064–2073.

[ref53] Zardoshti-Kermani M. Wheeler B. C. Badie K. Hashemi R. M. (1995). EMG feature evaluation for movement control of upper extremity prostheses. IEEE Trans. Rehabil. Eng. 3, 324–333. doi: 10.1109/86.481972

[ref54] Zeng C. Yang C. Zhong J. Zhang J. (2019). Encoding multiple sensor data for robotic learning skills from multimodal demonstration. IEEE Access 7, 145604–145613. doi: 10.1109/access.2019.2945484

[ref004] Zeng C. Yang C. Cheng H. Li Y. Dai S. L. (2020). Simultaneously encoding movement and sEMG-based stiffness for robotic skill learning. IEEE Transactions on Industrial Informatics 17, 1244–1252. doi: 10.1109/TII.2020.2984482

