# Supplementary material for: A multimodal human-robot sign language interaction framework applied in social robots
Source: Front Neurosci. 2023 Apr 11;17:1168888. doi: 10.3389/fnins.2023.1168888 (PMC10126358; doi:10.3389/fnins.2023.1168888)
Supplement: Supplementary file 1 [file Data_Sheet_1.docx]

Supplementary Material

# Supplementary Figures and Tables

## Supplementary Figures


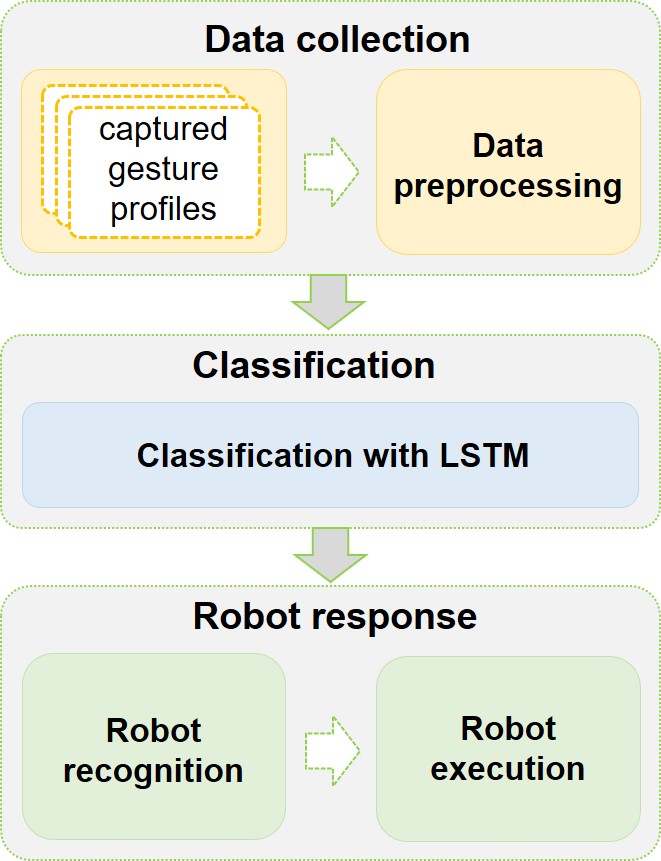


**Supplementary Figure 1.** The framework of the proposed multimodal HRI system.


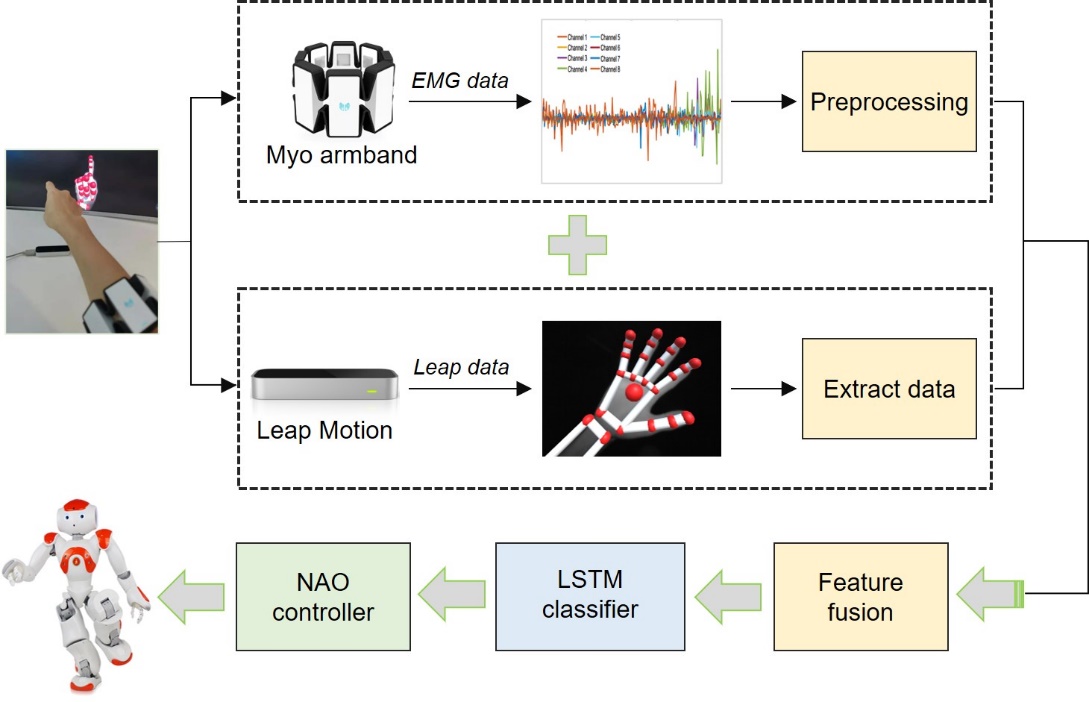


**Supplementary Figure 2.** An overall diagram of the HRI system.


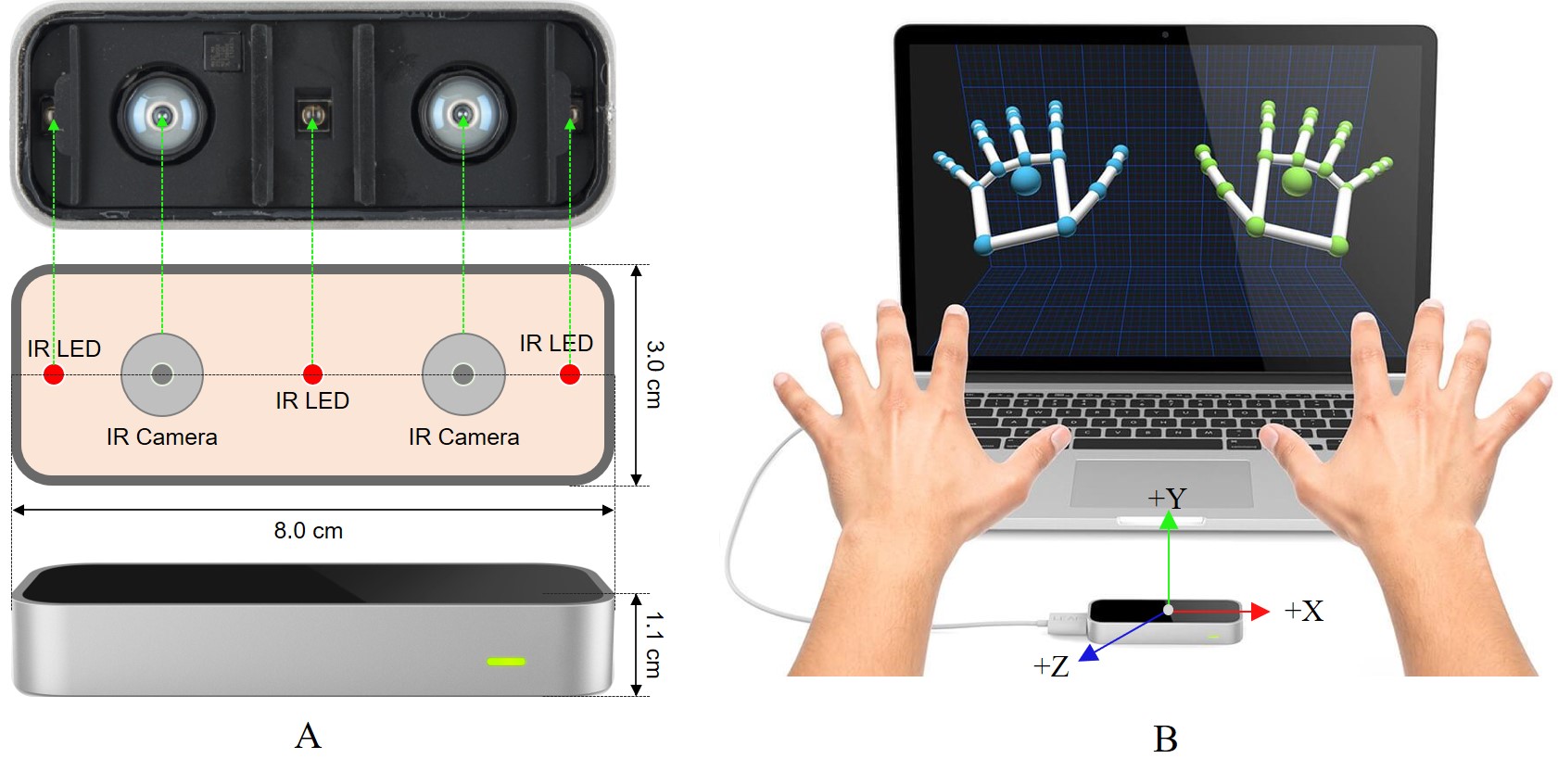


**Supplementary Figure 3.** The view of LMC. (A) Schematic view of LMC. (B) 3D view of human hand from LMC (Weichert et al., 2013).


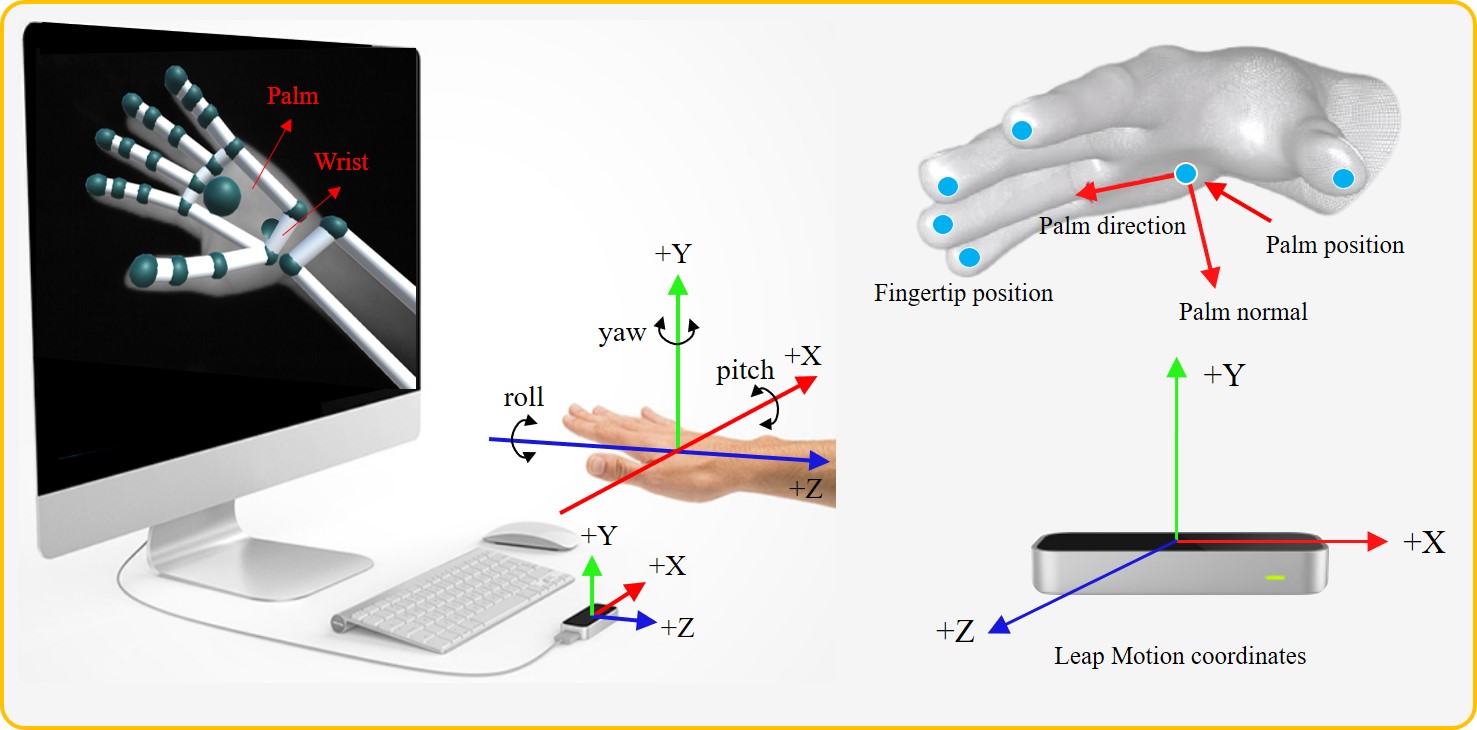


**Supplementary Figure 4.** The coordinate system of the Leap Motion sensor and diagram of the bone data detected by it.


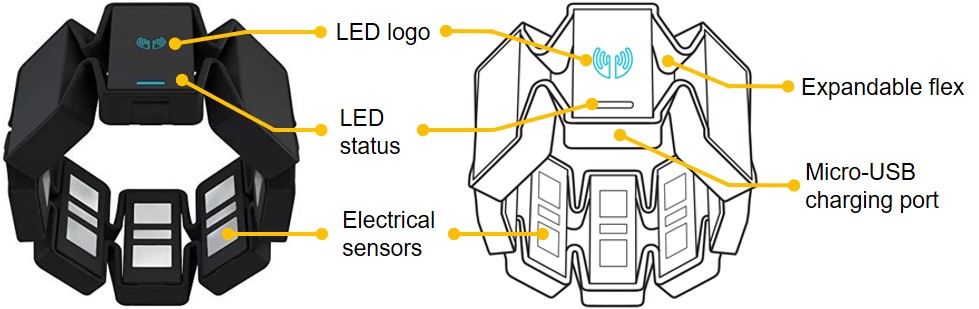


**Supplementary Figure 5.** The view of Myo armband.


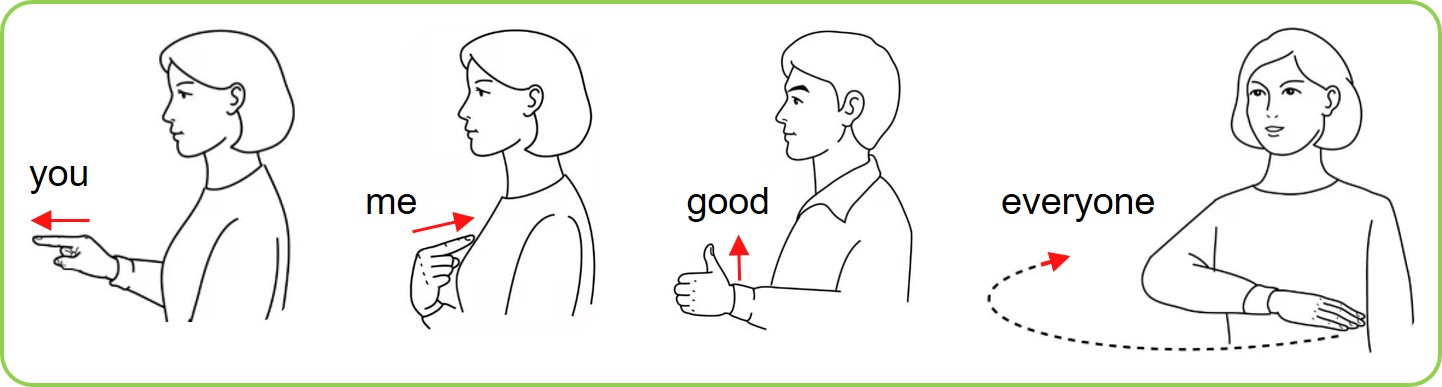


**Supplementary Figure 6.** Four kinds of CSL gestures.


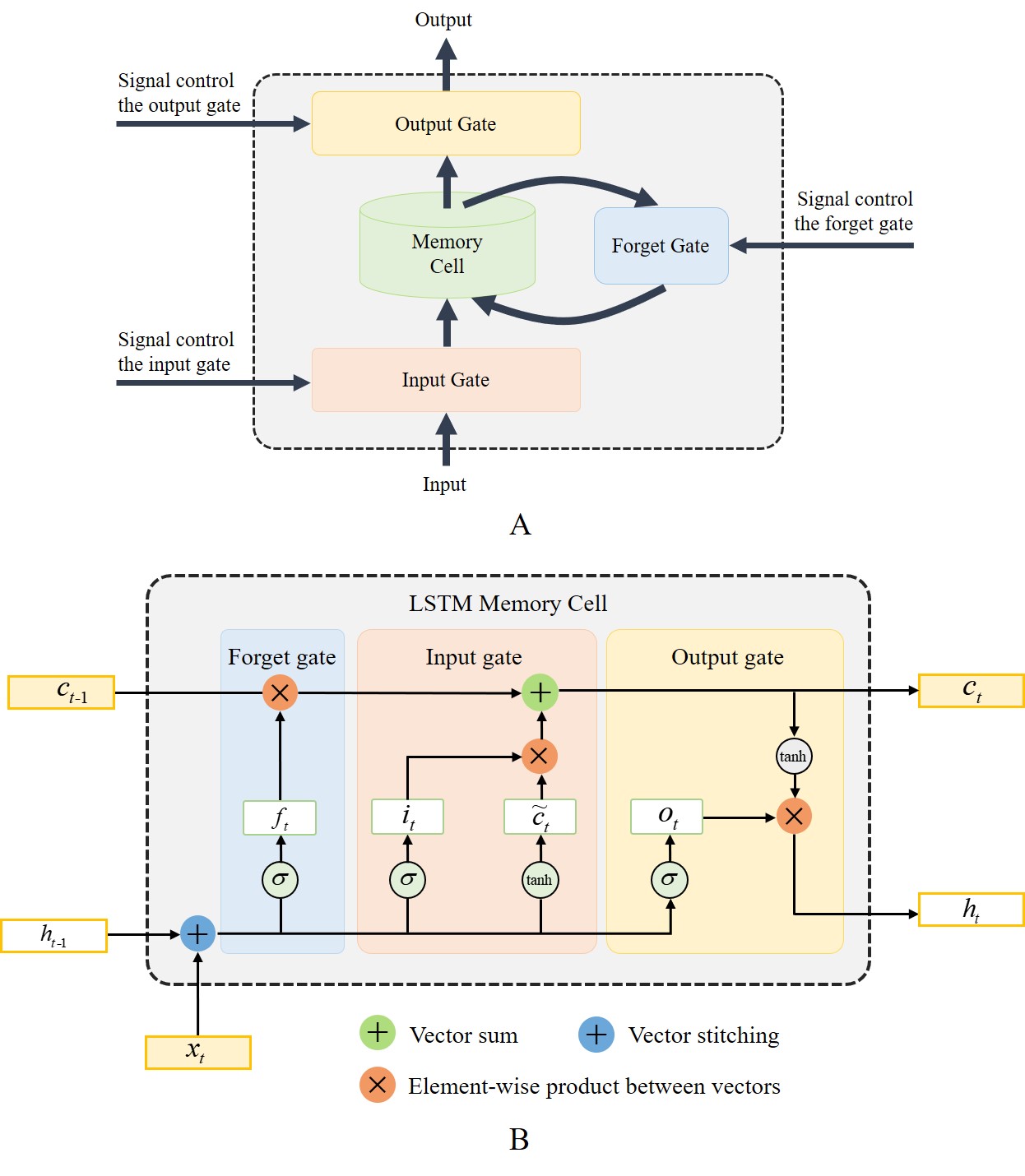


**Supplementary Figure 7.** The architecture of the LSTM network. (A) The composition of LSTM memory blocks. (B) The structure of LSTM memory cell.


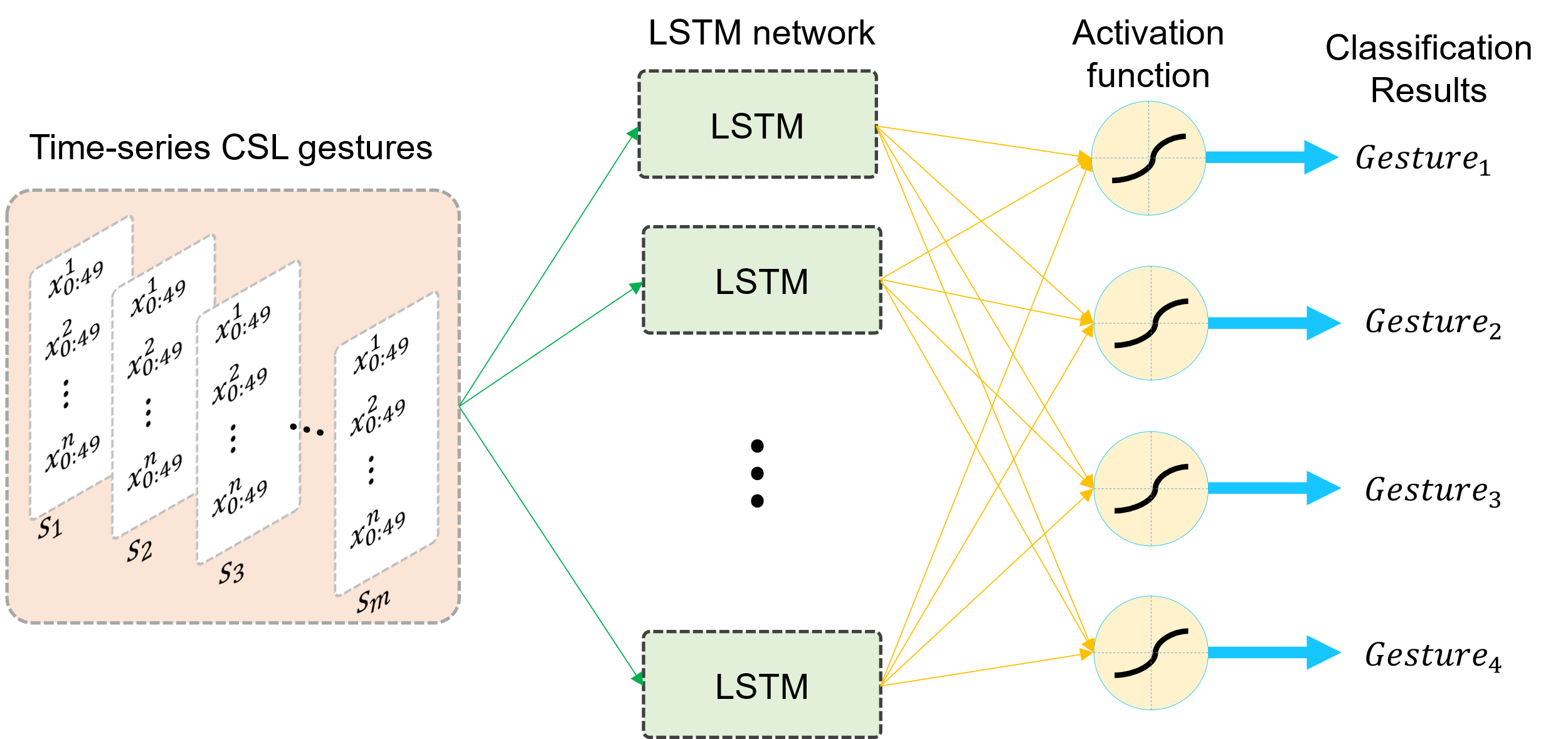


**Supplementary Figure 8.** The LSTM model used in the experiment.


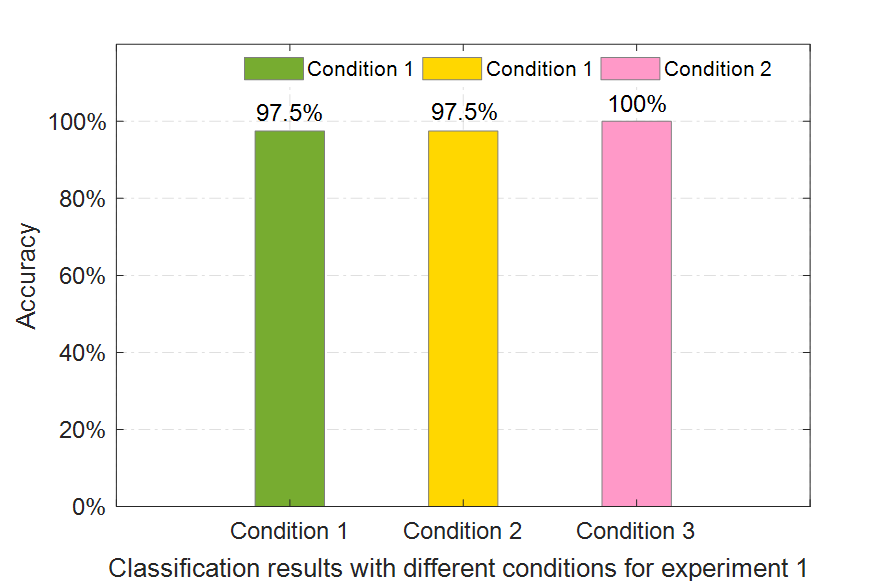


**Supplementary Figure 9.** Classification accuracies under three conditions for the first experiment.


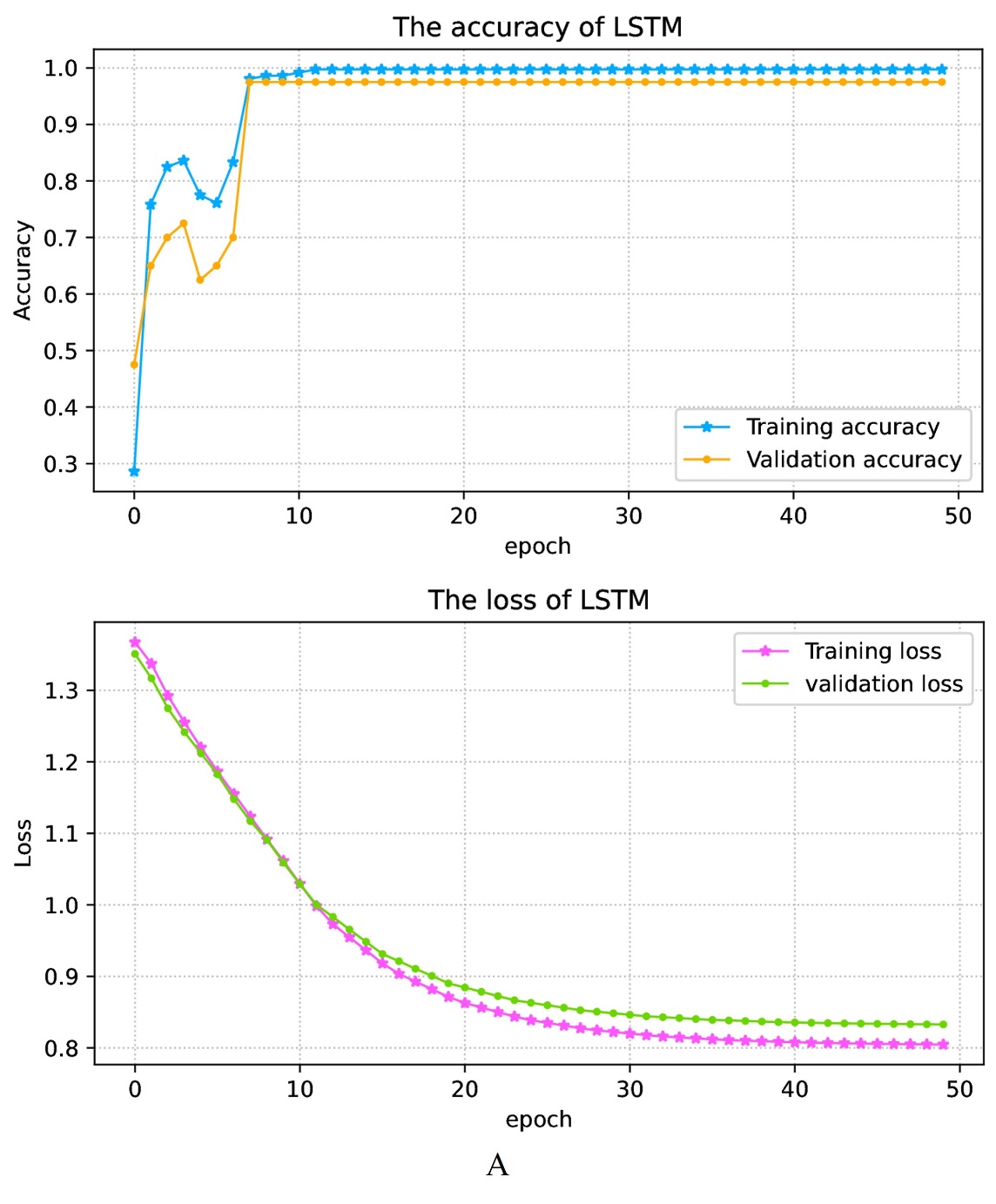

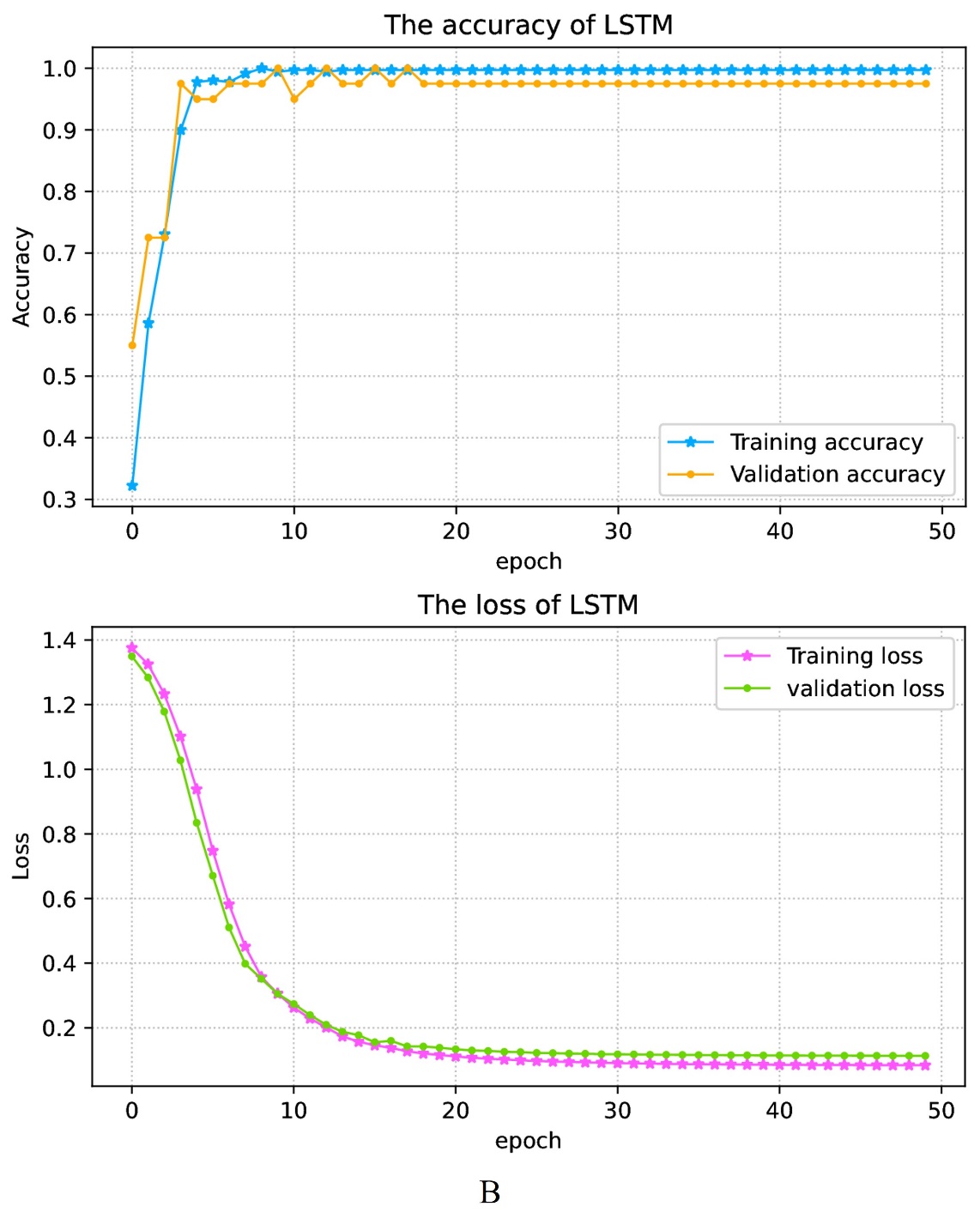

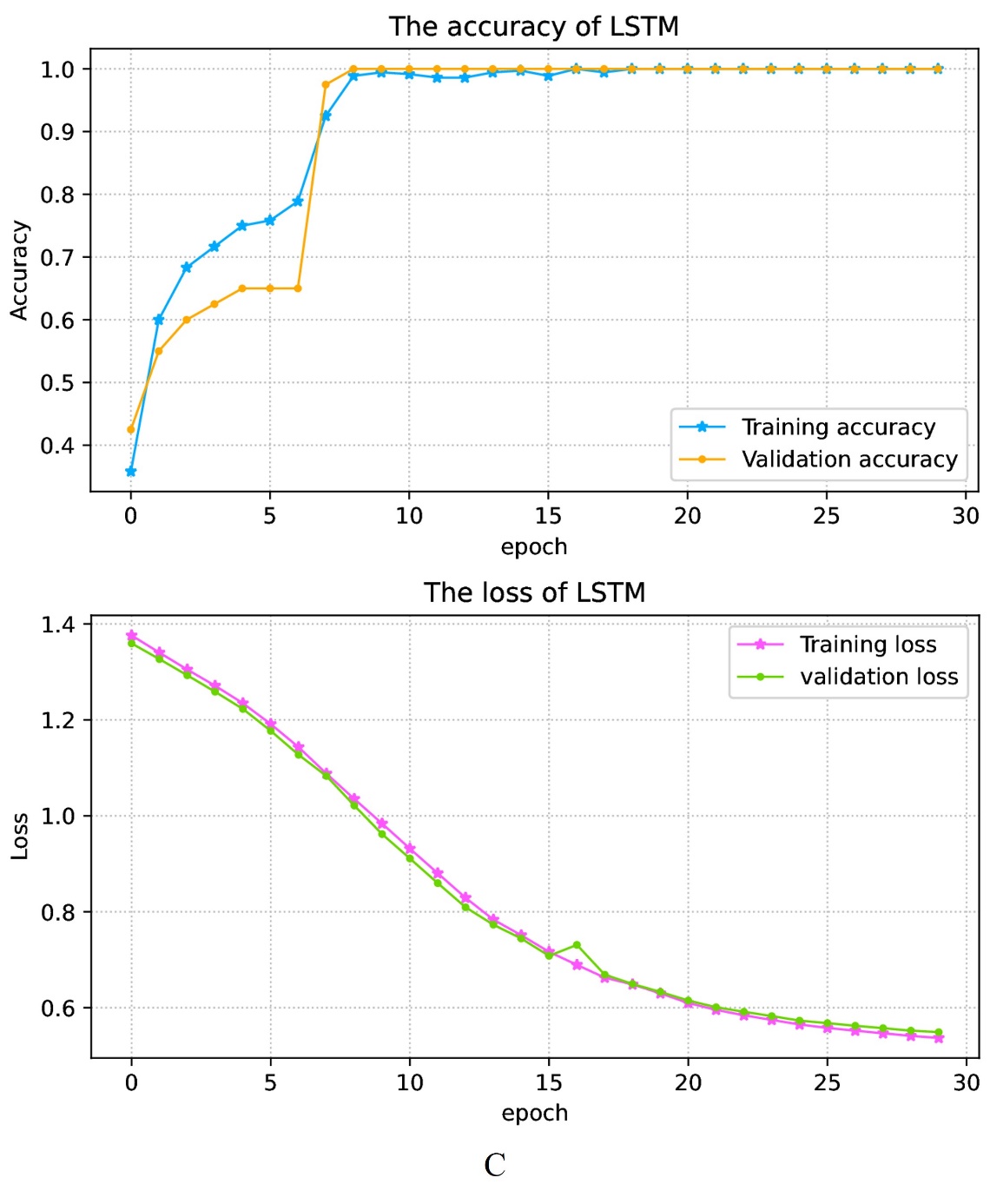


**Supplementary Figure 10.** The first experiment results under three conditions. (A) CSL gestures classification results under condition 1. (B) CSL gestures classification results under condition 2. (C) CSL gestures classification results under condition 3. In **Figures 10 (A-C)** the blue curves with star markers denote the recognition accuracy of the training set, and the yellow curves with circle markers denote the recognition accuracy of the testing set. The magenta and green curves are the loss values of the training sets and testing sets, respectively.


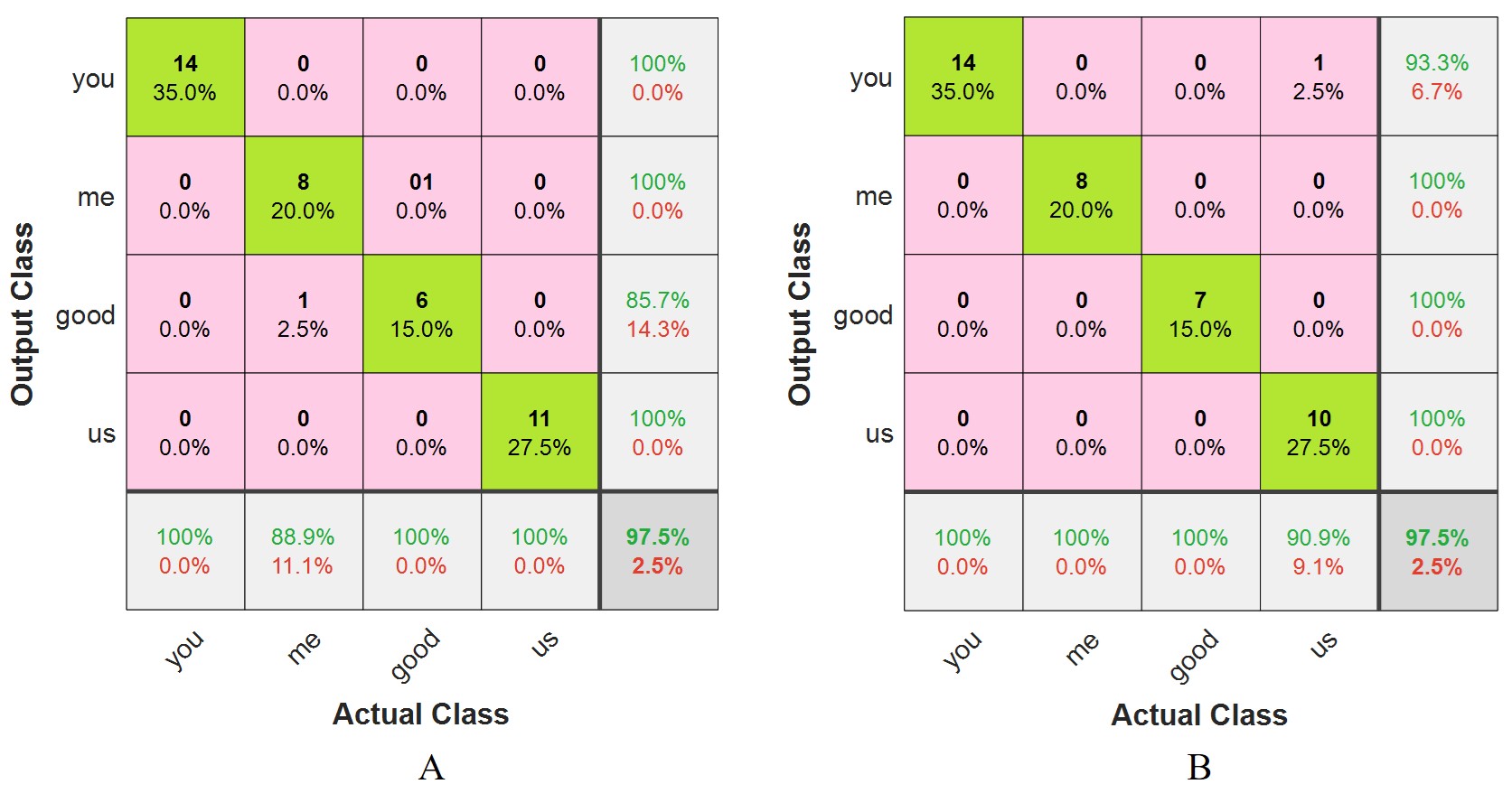


**Supplementary Figure 11.** The confusion matrices of the first experiment under conditions 1 and 2. (A) The confusion matrix under condition 1. (B) The confusion matrix under condition 2. In Figures 11 A and B, X-axis denotes the real sample labels, and the y-axis denotes the predicted sample labels. The top and bottom elements on the main diagonal filled with green color, respectively, represent the number and percentage of the samples that are correctly predicted. The top and bottom elements inside of each pink square, respectively, represent the number and percentage of wrong predicted samples. The top and bottom elements inside of lower and right light gray squares represent the prediction accuracy and error rate of corresponding samples.


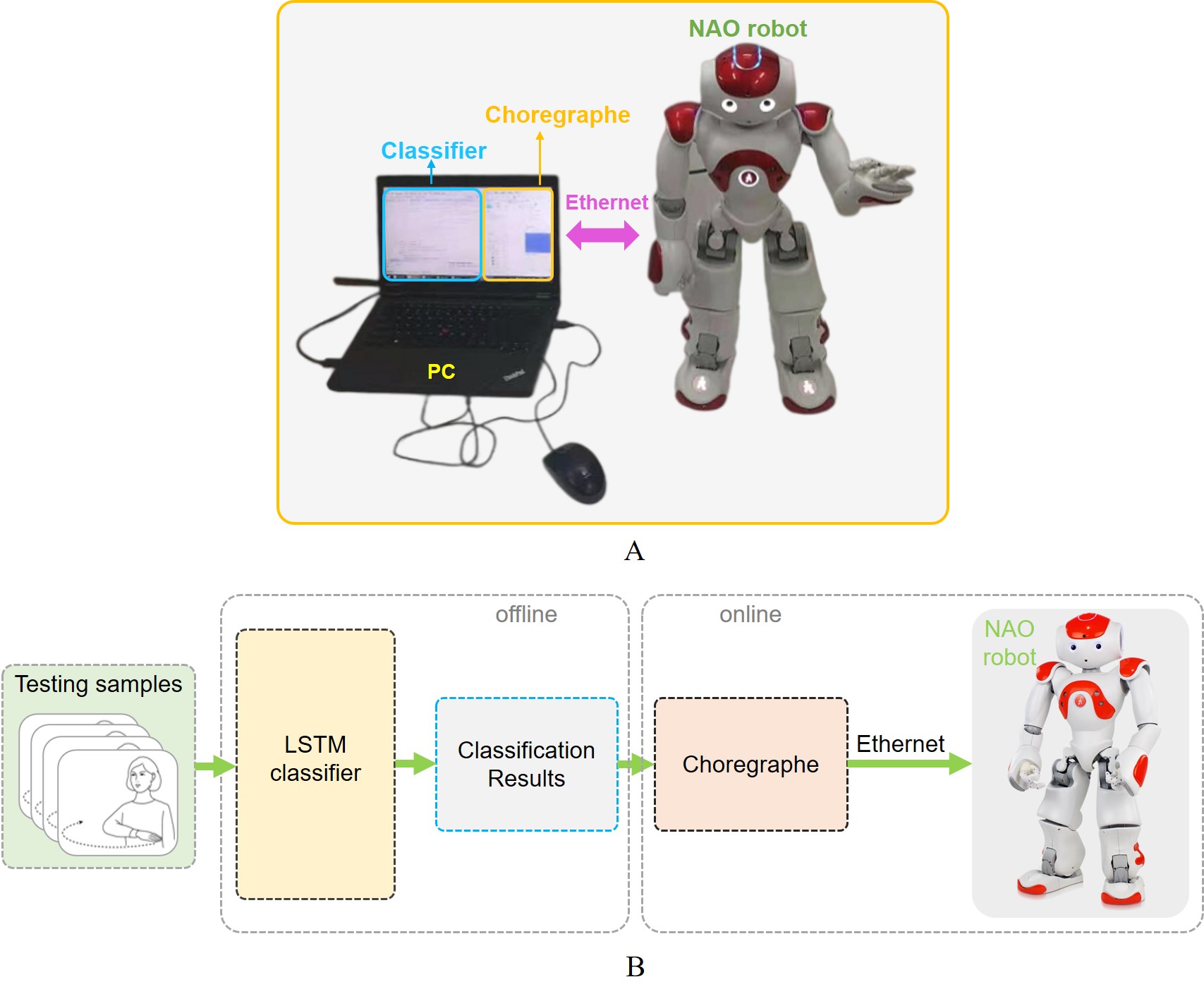


**Supplementary Figure 12.** The experimental system of the second experiment. (A) The experimental platform of the second experiment. (B) The experimental steps of the second experiment.


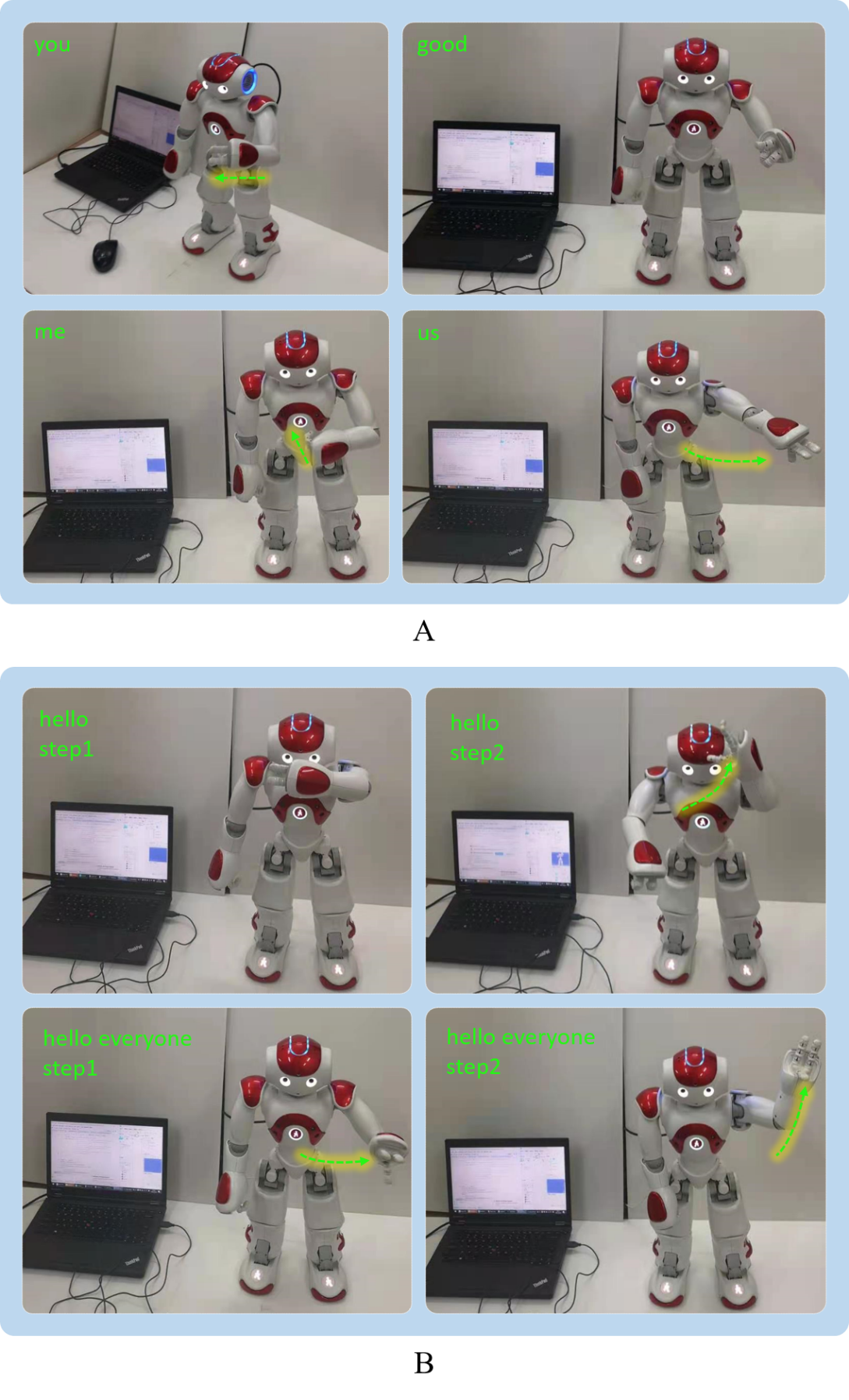


**Supplementary Figure 13.** The NAO robot interaction results of experiment 2. (A) Robot response results of the four singular hand gestures. (B) Robot response results of the two combination gestures.
